# Expression of spider silk protein in tobacco improves drought tolerance with minimal effects on its mechanotype

**DOI:** 10.1111/tpj.17213

**Published:** 2025-01-27

**Authors:** Shamitha Rao Morey‐Yagi, Yoichi Hashida, Masanori Okamoto, Masaki Odahara, Takehiro Suzuki, Chonprakun Thagun, Choon Pin Foong, Keiji Numata

**Affiliations:** ^1^ Biomacromolecules Research Team RIKEN Center for Sustainable Resource Science 2‐1 Hirosawa, Wako Saitama 351‐0198 Japan; ^2^ Laboratory for Biomaterial Chemistry, Department of Material Chemistry, Graduate School of Engineering Kyoto University Nishikyo‐ku Kyoto 615‐8510 Japan; ^3^ Laboratory of Crop Science, Faculty of Agriculture Takasaki University of Health and Welfare 54 Nakaorui‐machi Takasaki Gunma 370‐0033 Japan; ^4^ Plant Chemical Genetics Research Team RIKEN Center for Sustainable Resource Science 1‐7‐22, Suehiro, Tsurumi Yokohama Kanagawa 230‐0045 Japan; ^5^ Biomolecular Characterization Unit RIKEN Center for Sustainable Resource Science 2‐1 Hirosawa, Wako Saitama 351‐0198 Japan

**Keywords:** spider silk, drought tolerance, ABA, glycine‐rich, tensile strength, mechanotype

## Abstract

Spider silk, especially dragline silk from golden silk spiders (*Trichonephila clavipes*), is an excellent natural material with remarkable mechanical properties. Many studies have focused on the use of plants as biofactories for the production of recombinant spider silk. However, the effects of this material on the mechanical properties or physiology of transgenic plants remain poorly understood. Since glycine‐rich proteins play key roles in plants, we evaluated the effects of a glycine‐rich spider silk protein on plant mechanical properties (mechanotype) and physiology. We generated tobacco (*Nicotiana tabacum*) plants producing a nucleus‐ or plastid‐encoded partial component of dragline silk, MaSp1 (major ampullate spidroin‐1; *MaSp1*‐tobacco), containing six repetitive glycine‐rich and polyalanine tandem domains. MaSp1 accumulation had minimal effect on leaf mechanical properties, but improved drought tolerance. Transcriptome analysis of drought‐stressed *MaSp1*‐tobacco revealed the upregulation of genes involved in stress response, antioxidant activity, cellular metabolism and homeostasis, and phenylpropanoid biosynthesis. The effects of drought treatment differed between the nucleus‐ and the plastid‐encoded *MaSp1*‐tobacco, with the latter showing a stronger transcriptomic response and a higher total antioxidant status (TAS). Well‐watered *MaSp1*‐tobacco displayed elevated levels of the stress phytohormone ABA, leading to stomatal closure, reduced water loss, activation of stress response, and increased TAS. We show that the moderately enhanced ABA content in these plants plays a pivotal role in drought tolerance, alongside, ABA priming, which causes overall adjustments in multiple drought tolerance mechanisms. Thus, our findings highlight the potential of utilizing glycine‐rich spider silk proteins to enhance plant resilience to drought.

## Significance Statement

Expressing a glycine‐rich spider dragline silk (MaSp1) in tobacco improved its drought tolerance due to higher endogenous ABA content promoting stomatal closure, reducing water loss, increasing antioxidant status, and activating stress responses. These results lay the foundation for biomaterial production using agricultural biomass wastes from drought‐resilient crops.

## INTRODUCTION

The increase in agricultural and industrial activities due to the rising world population has led to an increase in the production of biomass wastes. Lack of proper biomass waste management has severe environmental consequences, especially in developing countries (Tripathi et al., 2019). Rising global temperatures due to anthropogenic activities are also leading to water scarcity, posing a major problem to agriculture in both developed and developing countries (Hanna et al., 2016). These issues represent the cause and effect of two of the greatest challenges faced by agriculture and the environment and demand the development of plant varieties with higher drought tolerance and stronger structural properties to enable food security, crop resilience, and the design of new biomaterials from plant biomass wastes.

Spider silk is a remarkable natural material with superior mechanical properties, including outstanding toughness, which surpasses the toughness of Kevlar and the strength of steel (on a weight basis) (Gosline et al., 1999; Vollrath & Knight, 2001). In addition to its impressive mechanical properties, spider silk, especially dragline silk from golden silk spider (*Trichonephila clavipes*), is light‐weight, biocompatible, and biodegradable, making it a promising candidate in the textile, biomedical (Salehi et al., 2020), and nanomaterial (Kiseleva et al., 2020) fields. Dragline (major ampullate) silk is composed of two spider fibroins (spidroins), major ampullate spidroin 1 (MaSp1) and major ampullate spidroin 2 (MaSp2), which contribute to the tensile strength and elasticity of dragline silks (Hinman et al., 2000; Hinman & Lewis, 1992; Xu & Lewis, 1990). Spidroins typically consist of three distinct domains: a glycine‐ and alanine‐rich repetitive central domain flanked by non‐repetitive N‐ and C‐terminal domains (Ayoub et al., 2007; Whittall et al., 2021). The basic units of the repeat domain consist of crystalline polyA or polyAG motifs (which impart strength) and less crystalline glycine‐rich (GGX or GPGXX) motifs (which impart elasticity) (Hayashi et al., 1999; Rising et al., 2005; Xu & Lewis, 1990).

Despite its wide range of applications, the large‐scale production of spider silk is unfortunately not feasible due to the difficulty in farming spiders, unlike its insect counterpart, the silkworm (*Bombyx mori*) (Koeppel & Holland, 2017). This limitation has led to attempts to heterologously produce it using yeast (*Saccharomyces cerevisiae*) (Fahnestock & Bedzyk, 1997), microbial (Fahnestock & Bedzyk, 1997; Foong et al., 2020; Prince et al., 1995; Xia et al., 2010), insect (Xu, Dong, et al., 2018), and plant systems (Menassa et al., 2004; Scheller et al., 2001). However, significant production levels in plant systems have not yet been achieved, casting doubt on the viability of using plants as an effective platform for recombinant spider silk production (Menassa et al., 2004; Whittall et al., 2021). One possible method for enhancing spider silk production in plants is plastid engineering, which enables high levels of transgene expression from multicopy plastid DNA (Maliga, 2004). However, since its development over a decade ago, no attempts have been made to increase the production of spider silk proteins in plants using this technology.

Current trends in biomaterials science include an increase in the engineering of new biocomposites with matrices made of living matter (Mohanty et al., 2018; Pugno & Valentini, 2019), largely due to their ecological sustainability, biocompatibility, and biodegradability (Mohanty et al., 2018). Plant materials are often used in biocomposites due to the natural hierarchy of the arrangement of plant cells, which are made up of various polymers (Fratzl & Weinkamer, 2007; Ganeriwala, 1990; Gibson, 2012). They exhibit a remarkable range of mechanical properties based on this cellular hierarchy, cell wall composition, and the components within the cell (Gibson, 2012). In addition to their natural hierarchy, a high level of anisotropy (varied mechanical properties in different orientations) also exists in plant tissues at the microlevel. However, despite this anisotropy, tobacco (*Nicotiana tabacum*) leaves exhibit isotropic (identical properties in different orientations) mechanical properties (Ganeriwala, 1990). Hence, the leaf, a natural composite, is an excellent material for comparing the mechanical performances of tobacco plants.

Glycine‐rich proteins (GRPs), characterized by high glycine contents, are found in various tissues of eukaryotes and exhibit a range of functions (Mousavi & Hotta, 2005). Similar to their structural roles in spider silk (Winkler & Kaplan, 2000), GRPs constitute major structural components of insect cuticles (Zhang et al., 2008) and plant cell walls (Czolpinska & Rurek, 2018; Mousavi & Hotta, 2005). Plant GRPs located in vascular tissues are believed to provide elasticity and tensile strength during vascular development (Cassab, 1998). In addition to their structural roles, GRPs also mediate responses to various biotic and abiotic stresses (Czolpinska & Rurek, 2018; Mousavi & Hotta, 2005). Overexpressing *GRP* genes in plants increases tolerance to abiotic stress (Czolpinska & Rurek, 2018) and improves germination and growth under stress conditions (Kim et al., 2007). The major mechanisms for GRP‐induced drought tolerance in rice (*Oryza sativa*) include their stabilization of the mRNA of genes encoding reactive oxygen species (ROS)‐scavenging enzymes and their modulation of the phenylpropanoid biosynthesis pathway due to the RNA binding ability of GRPs (Shim et al., 2021; Xu et al., 2022).

Plants respond to drought stress via various morphological, physiological, biochemical, cellular, and molecular mechanisms (Aroca, 2012). These mechanisms include osmotic adjustment, cellular water homeostasis, changes in fatty acid metabolism, modifications of RNA, DNA, and histones, phytohormone signaling pathways (abscisic acid [ABA], auxin, jasmonic acid, ethylene), and the scavenging of ROS by antioxidant enzymes and secondary metabolites, leading to enhanced drought tolerance (Aroca, 2012; Kim et al., 2015; Shinozaki & Yamaguchi‐Shinozaki, 2007). ABA is produced in plants under drought stress and plays an important role in eliciting drought stress responses (Aroca, 2012; Shinozaki & Yamaguchi‐Shinozaki, 2007). In addition, ABA is crucial for establishing and maintaining cross‐stress tolerance, that is, the improvement of plant performance under secondary stress due to pre‐exposure to primary stress (Liu, Able, & Able, 2022; Liu, Quan, & Bartels, 2022). Thus, priming plants with ABA during early growth elicits a cross‐stress memory that better equips plants for future drought stress by providing them with a faster, more efficient response to this stress (Liu, Quan, & Bartels, 2022). Plant stress memory encompasses changes in mechanisms involving epigenetics (RNA, DNA, and histone modifications); transcriptional regulation of transcription factor genes (*MYB, WRKY, basic leucine zipper* [*bZIP*]), plant hormone‐related genes (*Ethylene‐Response Factor* [*ERF*], *YUCCA8* [*YUC8*], *ARGOS*), transporter genes (Nitrate Transporter [*NRT*], *Plasma Membrane Intrinsic Protein 2* [*PIP2A*], *Sugars Will Eventually Be Exported Transporter1* [*SWEET1*]); various proteins (Peroxidase [*POX*], Heat‐Shock Protein [*HSP*], Late Embryogenesis Abundant [*LEA*], Proline/Serine‐Rich Protein [*PRP*]); and metabolites (phenylpropanoids, alkaloids, flavonoids, selenocompounds). The cumulative effect of these mechanisms is indispensable for drought tolerance (Liu, Able, & Able, 2022).

Here, we expressed the six‐repeat sequence of *MaSp1* (hereafter referred to as *MaSp1*) from *T. clavipes* (Prince et al., 1995) in the nucleus and plastids of tobacco (*Nicotiana tabacum*; hereafter collectively referred to as *MaSp1*‐tobacco) and evaluated its effect on plant mechanical properties (mechanotype) and physiology. The *MaSp1*‐tobacco showed higher drought tolerance and better recovery from drought than the wild‐type, with the plastid‐encoded *MaSp1*‐tobacco performing better than the nucleus‐encoded *MaSp1*‐tobacco, due to its higher antioxidant status. We show that the moderately elevated ABA content (compared with the non‐transformed control) in well‐watered *MaSp1*‐tobacco and ABA priming are responsible for this phenotype. Although *MaSp1* expression had no significant effect on plant mechanotype, we discuss possible solutions for the improved utility of spider silk‐expressing, naturally functionalized plant cells such as leaves and stems for structural applications. We also outline a simple, improved method for tensile testing of leaves, which can be used to evaluate the mechanical properties of leaves and/or leaf‐based biomaterials in the future.

## RESULTS

### Generation of *MaSp1* ‐expressing transgenic and transplastomic tobacco plants

**Figure 1:**
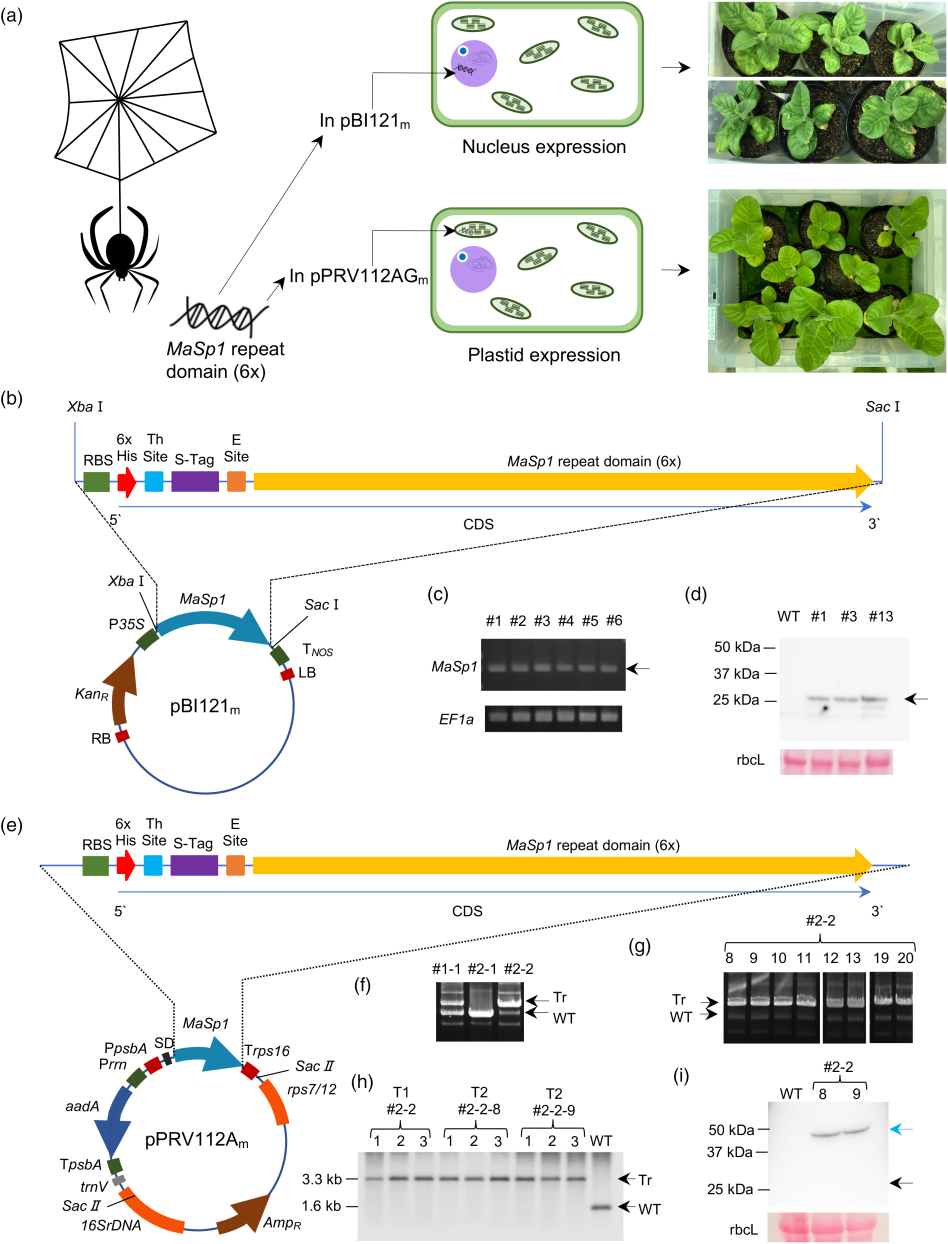
Generation of *MaSp1*‐expressing transgenic and transplastomic tobacco. (a) Approach for expressing a transgene encoding the MaSp1 repeat domain (6×) from the nuclear and plastid genomes of *N. tabacum*. (b) Schematic diagram of the modified vector pBI121_m_ used for the nuclear expression of *MaSp1* in tobacco, which was confirmed by (c) PCR genotyping and (d) immunoblotting of 7 μg of total soluble protein (TSP) per well from the T0 transgenic tobacco shoots using a monoclonal anti‐6 × His antibody. (e) Schematic diagram of the modified vector pPRV112A_m_ used for the plastid expression of *MaSp1* in tobacco, which was confirmed by (f) PCR genotyping of T0 and (g) T1 progeny, and (h) southern blotting of 10 μg genomic DNA from T1 and T2 progeny of #2–2 (hereafter *MaSp1*_*pla*_ #2–2) using a DNA probe targeted to the *trnV–16SrDNA* region following restriction digestion by *SacII*. Digestion with *SacII* yielded approximately 1.6 kb fragment in WT and approximately 3.3 kb fragment in the transplastomic lines with *P*_*psbA*_:*MaSp1:T*_*rps16*_ and *P*_*rrn*_:*aadA:T*_*psbA*_ insertion. Protein expression was confirmed by (i) immunoblotting of 22–23 μg of TSP per well from the T2 progeny of transplastomic tobacco shoots using a monoclonal anti‐6 × His antibody. CDS, coding sequence. Positive bands or signals of the expected sizes are indicated by black arrows. Dimerized MaSp1 in (h) is indicated by a blue arrow.

We introduced *MaSp1* (Figure S1a) along with the sequence encoding an N‐terminal His‐tag into the nuclear and plastid genomes of tobacco (*N. tabacum*) (Figure 1a) via *Agrobacterium tumefaciens*‐mediated transformation and biolistic gene delivery, respectively. To this end, we modified the vectors pBI121 (Chen et al., 2003) and pPRV112AG (Shiina et al., 2000) to replace the *ß‐GLUCURONIDASE* (*GUS*) and *enhanced Green Fluorescent Protein* (*eGFP*) sequences, respectively, with *MaSp1* (Figure S1b,c). We introduced the resulting constructs into the genome of the corresponding organelles.

Specifically, we introduced a *MaSp1* expression cassette (P_*CaMV35S*_:*MaSp1*:T_*NOS*_) and a kanamycin resistance gene into the nuclear genome (Figure 1b). We obtained 22 independent kanamycin‐resistant shoots, which we screened for the presence of *MaSp1* by PCR genotyping (representative image shown for 6 shoots); individuals with successful integration of this transgene showed a 750‐bp band on agarose gels (Figure 1c). Next, we examined MaSp1 accumulation in three PCR‐confirmed shoots by immunoblotting with an anti‐His‐tag antibody. All three shoots (#1, #3, and #13) showed a positive signal for MaSp1 (Figure 1d) with the expected size of approximately 25 kDa. Solely based on seed availability, we grew kanamycin‐resistant T1 seedlings of lines #1 and #3 (hereafter *MaSp1*_*nuc*_ #1 and *MaSp1*_*nuc*_ #3) alongside the non‐transformed control (WT) and the transplastomic plants described below for experiments. Following this, we confirmed the presence and integrity of MaSp1 by liquid chromatography coupled with tandem mass spectrometry (LC–MS/MS) analysis of protein bands excised from SDS‐PAGE gels corresponding to HisTrap‐purified fractions of *MaSp1*_*nuc*_ #1 (Dataset S1, Figure S2a,b).

For plastid transformation, we introduced *MaSp1* (P_*psbA*_:*MaSp1*:T_*rps16*_) and a spectinomycin/streptomycin resistance gene into the *trnV–rps7/12* intergenic region of the tobacco plastome (Figure 1e). We screened 112 shoots by PCR genotyping, looking for a 5.3‐kb band as evidence of successful transplastomic event relative to the 3.4‐kb band expected in WT (Figure 1f). We confirmed five shoots with transplastomic insertion of *MaSp1* by PCR (representative gel image for 2 out of 5 shoots shown in Figure 1f), but #2–2 (hereafter *MaSp1*_*pla*_ #2–2) was the only one that survived repeated rounds of culture and produced seeds. PCR of the T2 progeny (Figure 1g) and southern blotting of the T1 and T2 progeny (Figure 1h) confirmed the homoplastomic status of *MaSp1*_*pla*_ #2–2. Immunoblotting of the T1 progeny of the homoplastomic line *MaSp1*_*pla*_ #2–2 (Figure 1h) with an anti‐His‐tag antibody revealed a higher molecular weight His‐tagged protein of 50 kDa, which was almost twice the expected size (Figure S2c). Although *MaSp1*_*pla*_ #2–2‐11 and *MaSp1*_*pla*_ #2–2‐12 showed higher protein levels, they did not survive on soil to yield seeds. Hence, *MaSp1*_*pla*_ #2–2‐8 and *MaSp1*_*pla*_ #2–2‐9 were confirmed for MaSp1 expression (Figure 1i) and used for experiments solely based on seed availability. LC–MS/MS of the SDS‐PAGE bands from the HisTrap‐purified fractions of *MaSp1*_*pla*_ #2–2 corresponding to the expected size of 25 kDa confirmed the presence of MaSp1, but with its His‐tag cleaved (Dataset S1, Figure S2a,d). This was in agreement with the predicted peptide cleavage site of *MaSp1* that eliminates the first 45 amino acids of the N‐terminal sequence consisting of the His‐tag (Figure S2e,f).

To confirm the localization of MaSp1 in chloroplasts, we performed immunoblotting of the soluble protein fraction from isolated chloroplasts, which also showed a signal of 50‐kDa (Figure S3a). LC–MS/MS identification of the 50‐kDa band was inconclusive due to the interference of RbcL running at the same size. However, this larger protein was present only in *MaSp1*_*pla*_ #2–2 chloroplasts, but not in WT or *GFP*_*pla*_ chloroplasts (Figure S3a [right panel]), suggesting that MaSp1 might dimerize within plastids. The protein yields ranged from 0.4% to 0.8% MaSp1/total soluble protein (TSP) in *MaSp1*_*nuc*_ (Figure S3b) and from 0.5% to 0.7% MaSp1/TSP in *MaSp1*_*pla*_ (Figure S3c). We grew T1 seedlings from *MaSp1*_*pla*_ #2–2 alongside WT, *MaSp1*_*nuc*_ #1, and *MaSp1*_*nuc*_ #3 seedlings for experiments, except for experiments pertaining drought stress at ψ_w_ − 0.63 MPa and growth analysis, where T2 progeny of *MaSp1*_*pla*_ #2–2 (*MaSp1*_*pla*_ #2–2‐8 and *MaSp1*_*pla*_ #2–2‐9) were evaluated.

### Growth characteristics of *MaSp1* ‐tobacco

**Figure 2:**
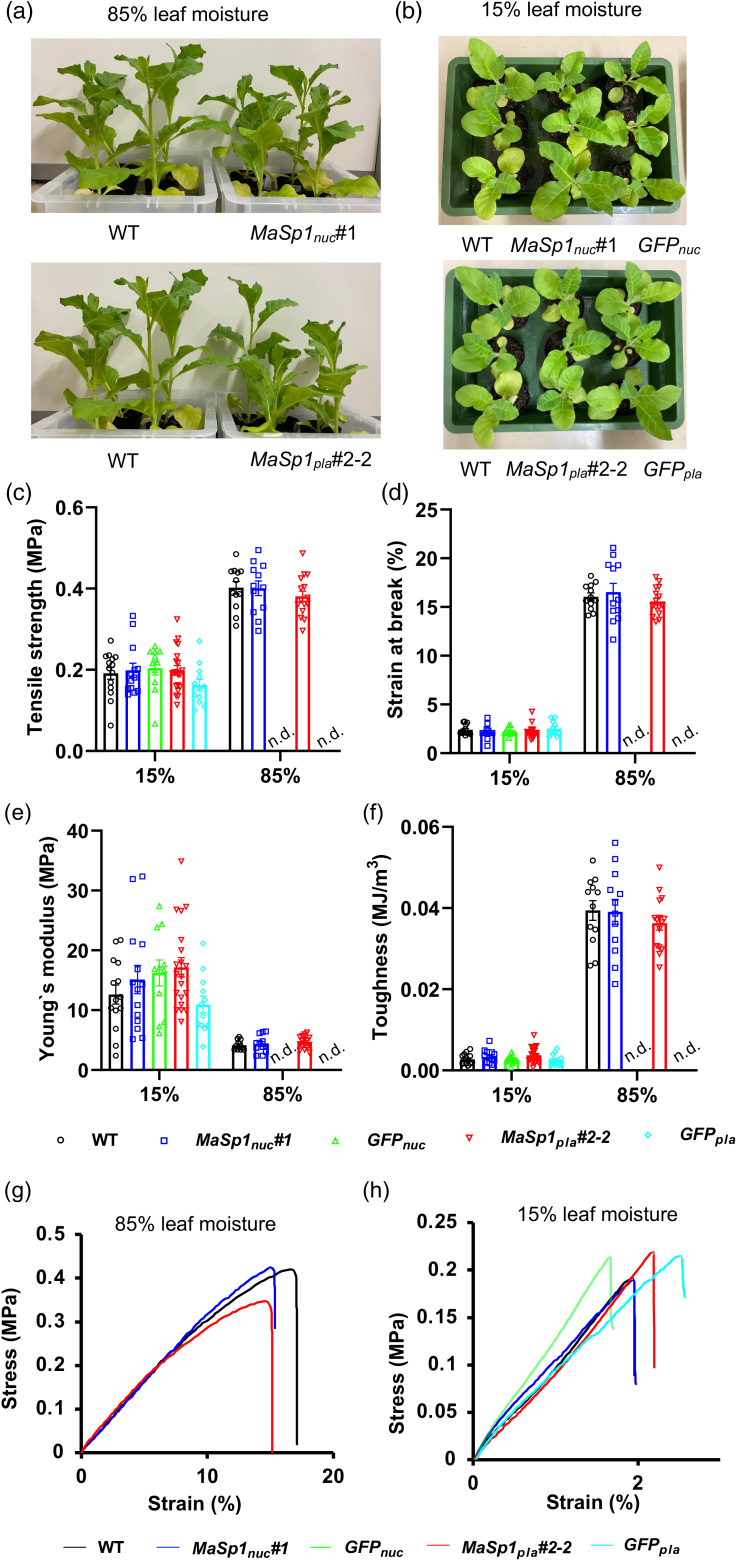
Tensile test of leaves of WT, *MaSp1*_*nuc*_ #1, *GFP*_*nuc*_, *MaSp1*_*pla*_ #2–2, and *GFP*_*pla*_ plants with varying moisture contents. Representative photographs of plants used for the tensile test at (a) 85% and (b) 15% leaf moisture. (c) Tensile strength, (d) strain at break, (e) Young's modulus, and (f) toughness of leaves at 85% and 15% leaf moisture content. Stress–strain curves of representative leaf sections of WT, *MaSp1*_*nuc*_ #1, *GFP*_*nuc*_, *MaSp1*_*pla*_ #2–2, and *GFP*_*pla*_ with (g) 85% and (h) 15% leaf moisture contents. Data represent means ± SEM of *n* = 12 to 20 leaf sections from 4 individual plants. No significant difference in values between the genotypes was observed using one‐way ANOVA (Tukey's test). Representative data closest to the average values for each line are shown in the stress–strain curves. n.d. indicates no data.

The single‐shoot dry weights (DW) of WT, *MaSp1*_*nuc*_, and *MaSp1*_*pla*_ plants grown in soil were measured at 8 days after sowing (DAS), 15 DAS, 22 DAS, 37 DAS, and 52 DAS (Figure S4a). There were no differences in the DW between the genotypes until 22 DAS (Figure S4b). However, by 37 DAS *MaSp1*_*pla*_ displayed lower DW compared with the WT (Figure S4c), and by 52 DAS, both *MaSp1*_*nuc*_ and *MaSp1*_*pla*_ showed lower biomass compared with the WT (Figure S4a). Thus, *MaSp1*‐tobacco plants were smaller than the WT in higher growth stages, which is consistent with its appearance at 72 DAS (Figure 2a).

### Leaf tensile properties of *MaSp1* ‐tobacco at varying leaf moisture levels

Tensile testing is a materials science test that evaluates the strength and elasticity of a material by applying a controlled force until the material completely fails or breaks. To determine whether *MaSp1*‐tobacco had enhanced tensile properties, we used fresh leaves at 85% leaf moisture (w/w) and moisture‐conditioned leaves at 15% leaf moisture (w/w) (Figure S5) from WT, *MaSp1*_*nuc*_ #1, GFP_nuc_, *MaSp1*_*pla*_ #2–2, and *GFP*_*pla*_ plants (Figure 2a,b) to prepare samples for tensile tests (Figure S6). To account for the effects of repeated culture and regeneration on the mechanical properties of leaves, we used the vector controls, *GFP*_*nuc*_ and *GFP*_*pla*_ bearing *eGFP* instead of *MaSp1* in pBI121_m_ (Figure S7a) and pPRV112A_m_ (Figure S7b), respectively.

We observed no significant differences in tensile strength (Figure 2c), strain at break (Figure 2d), Young's modulus (a measure of the stiffness of a material; Figure 2e), and toughness (Figure 2f) between WT and *MaSp1*‐tobacco leaf samples with either 85% or 15% leaf moisture content. In leaf samples with 15% leaf moisture content, Young's modulus tended to be higher in *MaSp1*_*pla*_ #2–2 than the vector control *GFP*_*pla*_ (Figure 2e) due to the tendency of *GFP*_*pla*_ to have lower tensile strength (Figure 2c) and higher strain at break (Figure 2d). Furthermore, *MaSp1*_*nuc*_ #1 and *MaSp1*_*pla*_ #2–2 leaves tended to be tougher than the WT and their respective vector controls *GFP*_*nuc*_ and *GFP*_*pla*_ (Figure 2f). The leaf area (Figure S8a) and leaf thickness (Figure S8b) of leaf samples, and the height (Figure S8c) and DW of plants (Figure S8d) used for the tensile test were not significantly different between the *MaSp1*‐tobacco lines and their respective *GFP* controls, the DW however, tended to be lower than the WT (Figure S8d).

The stress–strain curves of all leaf samples consistently showed an elastic–plastic tendency at 85% leaf moisture (Figure 2g), with a higher strain at break (Figure 2d) and almost twice the tensile strength (Figure 2c) compared with samples with 15% leaf moisture. Leaf samples with 15% leaf moisture failed in the elastic region of the curve, that is, before reaching the plastic region of the curve (Figure 2h), which was otherwise observed in samples with 85% leaf moisture. These results reflect the true nature of tobacco leaves: they are soft and elastic when hydrated (at 85% leaf moisture) and are stiff and break easily upon losing moisture, that is, at 15% leaf moisture. However, the effect of MaSp1 on leaf tensile properties remained limited.

### Drought stress recovery of *MaSp1* ‐tobacco

**Figure 3:**
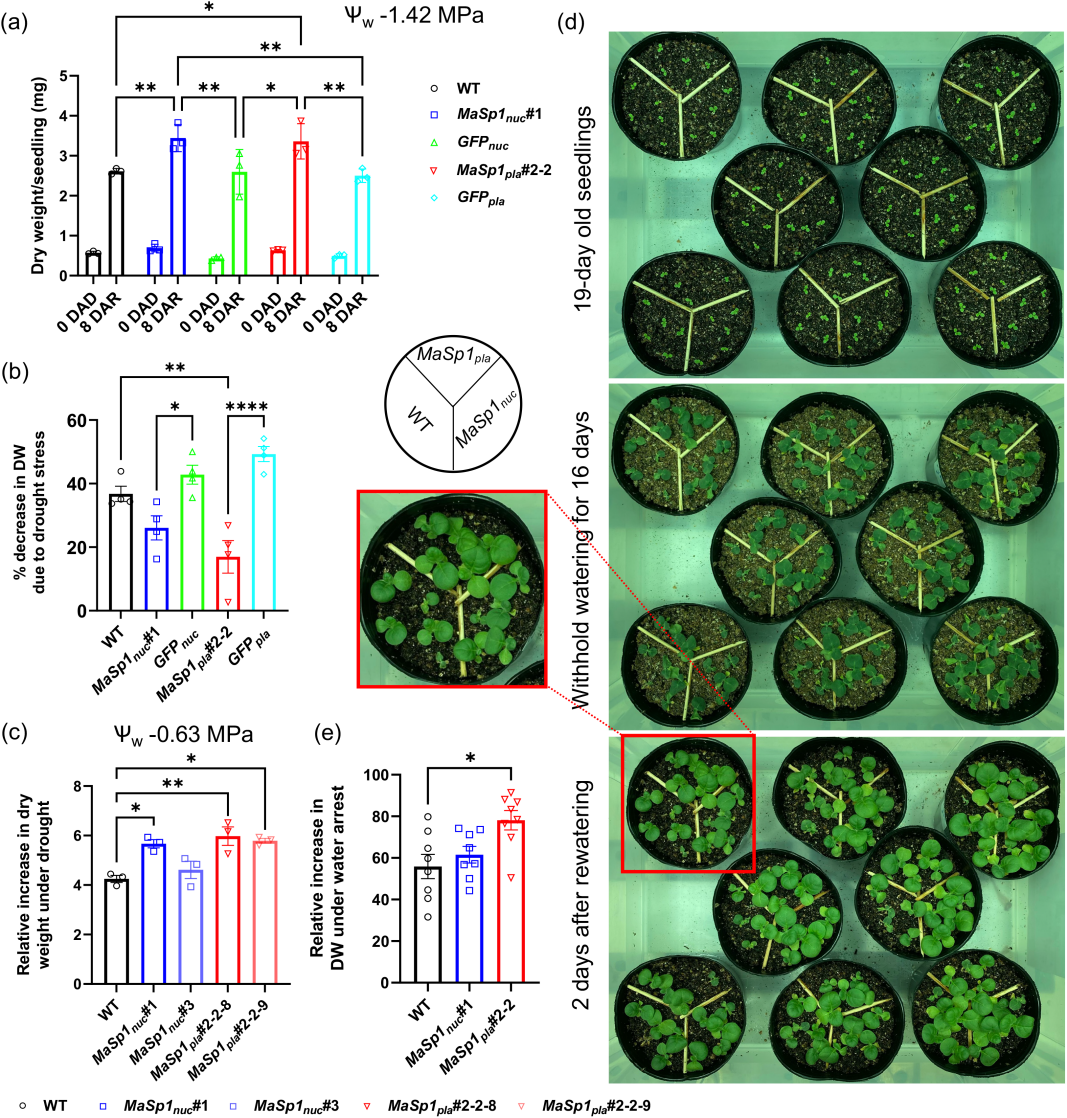
Drought tolerance and recovery of *MaSp1*‐tobacco. *MaSp1*‐tobacco were grown in (a–c) PEG‐containing medium at (a), (b) ψ_w_ − 1.42 MPa and (c) ψ_w_ − 0.63 MPa and in (d), (e) soil. (a) Single seedling dry weight of WT, *MaSp1*‐expressing plants (#1_nuc_ and #2‐2_pla_), and their corresponding *GFP* controls (*GFP*_*nuc*_ and *GFP*_*pla*_) before drought treatment (0 days after drought induction [DAD]) and 8 days after recovery (DAR) (*n* = 12 to 40). (b) % decrease in seedling dry weight due to drought at 2 DAR, obtained by comparing the weights of the respective lines grown under unstressed conditions for the same period of time (*n* = 12–40). (c) Increase in single seedling dry weight during drought at 9 DAD compared with 0 DAD (*n* = 12–16). (d) Growth of 19‐day‐old WT and *MaSp1*‐expressing plants (#1_nuc_ and #2‐2_pla_) on soil under 16 days of water withholding treatment and after recovery. The magnified image shows increased growth and recovery of *MaSp1*‐expressing plants under water withholding treatment from a representative pot. (e) Increase in seedling dry weight under water withholding treatment at 2 days after rewatering relative to the weights of the 18‐day‐old seedlings before drought initiation (*n* = 40). Data represent means ± SEM. Asterisks indicate significant differences at * *P* < 0.05, ** *P* < 0.01 and *** *P* < 0.001, as determined by one‐way ANOVA (Tukey's test).

Hydrophilic proteins, such as LEAs and other hydrophilins play protective roles in plants under water deficit (Battaglia et al., 2008). The substantial hydrophobicity of MaSp1 prompted us to examine its effect on the response of *MaSp1*‐tobacco to water deficit in both PEG‐infused growth medium and soil. The low water potential of PEG allowed us to simulate the drought stress conditions (Verslues et al., 2006). We grew *MaSp1*_*nuc*_ #1 and *MaSp1*_*pla*_ #2–2 plants alongside the WT and their respective vector controls, *GFP*_*nuc*_ and *GFP*_*pla*_ in MS medium for 13 days, following which they were moved to polyethylene glycol (PEG)‐infused growth medium (ψ_w_ − 1.42 MPa) for 7 days, and then rescued by transfer to MS medium. The DWs at 8 days after recovery (DAR) were significantly higher in *MaSp1*_*nuc*_ #1 and *MaSp1*_*pla*_ #2–2 than the vector controls and the WT (Figure 3a).

To examine the decrease in DW during the 7‐day drought period (ψ_w_ − 1.42 MPa), we compared the DWs of drought‐stressed seedlings at 2 DAR with the DWs of unstressed seedlings in MS medium for the respective genotypes at the same growth stage (22 DAS). Both *MaSp1*_*nuc*_ #1 and *MaSp1*_*pla*_ #2–2 exhibited improved growth after recovery from drought stress, compared with the WT and their respective vector controls (Figure S9). However, under unstressed conditions, *MaSp1*_*pla*_ #2–2 displayed dry weights comparable to WT but lower than its vector control *GFP*_*pla*_, while *MaSp1*_*nuc*_ #1 showed growth comparable to its vector control *GFP*_*nuc*_, but greater than the WT (Figure S9). These data indicate that drought treatment had significantly milder effects on growth in both *MaSp1*_*nuc*_ #1 and *MaSp1*_*pla*_ #2–2 compared with the vector controls, wherein only *MaSp1*_*pla*_ #2–2 showed significant differences from the WT (Figure 3b). To explore this further, we grew 14‐day‐old *MaSp1*_*nuc*_ #1, *MaSp1*_*nuc*_ #3, *MaSp1*_*pla*_ #2–2‐8, and *MaSp1*_*pla*_ #2–2‐9 seedlings alongside the WT in PEG‐infused growth medium (ψ_w_ − 0.63 MPa) for 9 days and monitored their growth. *MaSp1*_*nuc*_ #1, *MaSp1*_*pla*_ #2–2‐8, and *MaSp1*_*pla*_ #2–2‐9 showed significantly higher growth than the WT, while *MaSp1*_*nuc*_ #3 displayed comparable growth to the WT (Figure 3c). Thus, the impact of low water potential treatments (ψ_w_ − 1.42 MPa and ψ_w_ − 0.63 MPa) on plant growth was reduced in *MaSp1*‐tobacco compared with the WT, with *MaSp1*_*pla*_ exhibiting a more resilient phenotype than the *MaSp1*_*nuc*_.

To monitor their recovery from drought, we transferred 16‐day‐old WT, *MaSp1*_*nuc*_ #1, and *MaSp1*_*pla*_ #2–2 seedlings to well‐watered soil, withheld watering for 16 days, and provided the plants with water for recovery (Figure 3d). To examine the drought response of these plants, we compared their DWs at recovery (2 DAR) and at the start of the experiment (16 DAS). *MaSp1*_*pla*_ #2–2 showed significantly higher growth when subjected to water withholding treatment compared with the WT (Figure 3d [magnified] and e), while *MaSp1*_*nuc*_ #1 displayed a tendency toward better growth than the WT upon recovery (Figure 3d [magnified] and *3e*). Thus, *MaSp1*_*pla*_ demonstrated faster recovery from drought in soil compared with WT.

### Examining the effects of drought on the transcriptome of *MaSp1* ‐tobacco

**Figure 4:**
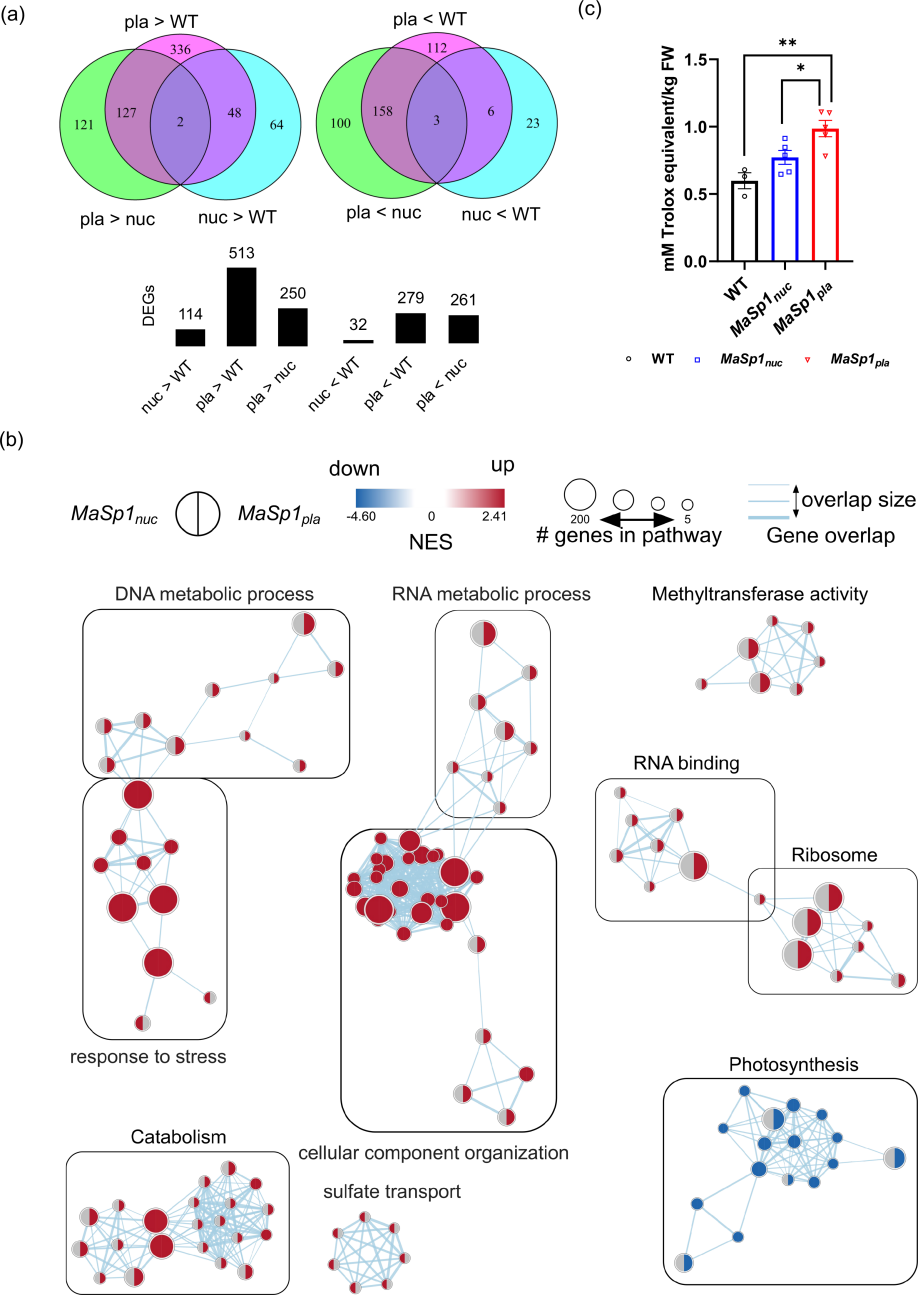
RNA‐seq and total antioxidant status of *MaSp1*‐tobacco grown at ψ_w_ − 0.63 MPa. (a) Differentially expressed genes (DEGs) with more than twofold differences in expression between drought‐stressed WT, *MaSp1*_*nuc*_, and *MaSp1*_*pla*_, where a higher number of DEGs was observed in *MaSp1*‐expressing plants with *MaSp1*_*pla*_ > *MaSp1*_*nuc*_. (b) Gene set enrichment analysis (GSEA) of 10 highly enriched GO terms with more than twofold differences relative to the WT. (c) Higher total antioxidant status of *MaSp1*‐expressing plants (*MaSp1*_*pla*_ > *MaSp1*_*nuc*_) compared with WT for *n* = 12–20 plants, where each sample point corresponds to the average value of four plants from different culture plates. Data represent means ± SEM. Asterisks indicate significant differences at *P < 0.05 and ** *P* < 0.01, as determined by one‐way ANOVA (Tukey's test). RNA‐seq data were obtained for pooled RNA from *n* = 8 plants per genotype.

To elucidate the mechanism of drought tolerance in plants expressing *MaSp1*, we performed transcriptome deep sequencing (RNA‐seq) of WT, *MaSp1*_*nuc*_ (#1 and #3), and *MaSp1*_*pla*_ (#2–2‐8 and #2–2‐9) plants grown under PEG‐induced drought conditions (ψ_w_ − 0.63 MPa) and identified differentially expressed genes (DEGs) with more than twofold differences in expression between the genotypes (Figure 4a; Table S1). We identified more DEGs in *MaSp1*_*pla*_ compared with *MaSp1*_*nuc*_ and the WT in response to drought (Figure 4a, bottom panel and Table S1). Gene set enrichment analysis (GSEA) of Gene Ontology (GO) terms identified “response to stress,” “cellular component organization,” and “catabolism” as the commonly upregulated functional gene clusters and “photosynthesis” as the commonly downregulated gene cluster in *MaSp1*‐tobacco (Figure 4b). Other gene clusters such as the “DNA and RNA metabolic process”, “methyltransferase activity,” “RNA binding,” “ribosome,” and “sulfate transport” were exclusively upregulated in *MaSp1*_*pla*_ (Figure 4b).

### Increased antioxidant properties of *MaSp1* ‐tobacco

The functional gene cluster “response to stress” (Figure 4b) consists of genes belonging to the GO terms peroxidase activity, antioxidant activity, oxidoreductase activity, heme binding, tetrapyrrole binding, and response to oxidative stress (Figure S10a). Additionally, GSEA of Kyoto encyclopedia of genes and genomes (KEGG) terms revealed the upregulation of genes related to “phenylpropanoid biosynthesis” and “phenylalanine metabolism” in *MaSp1*‐tobacco, whereas genes in the KEGG terms “flavonoid and isoquinoline alkaloid biosynthesis,” “selenocompound metabolism,” “cysteine, methionine, and tyrosine metabolism” were only upregulated in *MaSp1*_*pla*_ (Figure S10b).

These GO and KEGG terms pointed toward the mitigation of oxidative stress as a key mechanism for MaSp1‐induced drought tolerance. Hence, we evaluated the total antioxidant status (TAS) of drought‐stressed *MaSp1*‐tobacco and WT plants. TAS values were significantly higher in *MaSp1*_*pla*_ compared with *MaSp1*_*nuc*_ and WT plants, while that of *MaSp1*_*nuc*_ tended to be higher than the WT (Figure 4c). These results are consistent with the results of GSEA‐KEGG analysis which displayed the upregulation of genes involved in the biosynthesis of antioxidant secondary metabolites, such as flavonoids, alkaloids, and selenocompounds, exclusively in *MaSp1*_*pla*_ (Figure S10b).

### ABA content, stomatal regulation, and TAS in well‐watered *MaSp1* ‐tobacco

**Figure 5:**
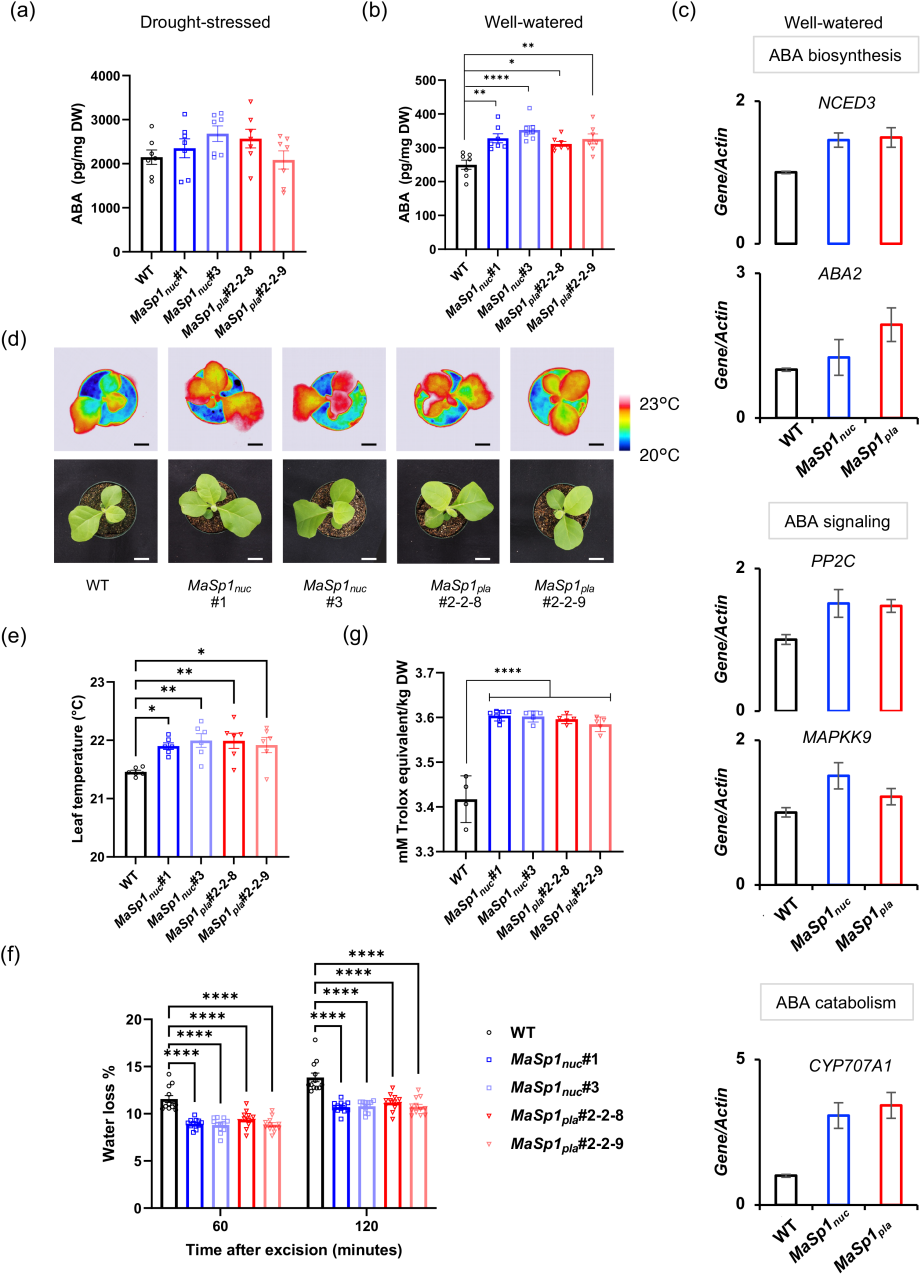
Moderate increase in ABA content of well‐watered *MaSp1*‐tobacco leads to drought tolerance. ABA content in the shoots of WT and *MaSp1‐tobacco* under (a) drought stress (14‐day‐old seedlings in soil–water withheld for 22 days without recovery), and (b) in well‐watered conditions (37‐day‐old seedlings in soil) from *n* = 7 groups of 3 plants each. (c) RT‐qPCR of genes involved in (*top panel*) ABA biosynthesis (9‐cis‐epoxycarotenoid dioxygenase (*NCED3*) and Xanthoxin dehydrogenase (*ABA2*)), (*middle panel*) ABA signaling (Serine threonine protein phosphatase 2C (*PP2C*) and Mitogen‐activated protein kinase kinase (*MAPKK9*)) and (*bottom panel*) ABA catabolism (cytochrome P450 (*CYP707A1*)), expressed as relative abundance to *Actin* in shoots from *n* = 15 plants, where the gene/*Actin* ratio for WT was set to 1. (d) Approximately 30‐day‐old plants were observed by infrared camera at 23°C. The top panel shows infrared images, and the bottom shows photos of plants. Bars indicate 2 cm. (e) The surface temperature of the leaves was analyzed using an infrared camera. Data were obtained from six independent plants. (f) Total antioxidant status (TAS) in shoots from non‐stressed *MaSp1*_*nuc*_ and *MaSp1*_*pla*_ and the WT (*n* = 4–7 plants). (g) Leaves from approximately 30‐day‐old plants were detached and placed at 23°C, and their weights were measured at the beginning, 1 and 2 h later. Data were obtained from 12 independent plants and represent means ± SEM. Asterisks indicate significant differences at * *P* < 0.05, ** *P* < 0.01, and **** *P* < 0.0001 using one‐way ANOVA (Tukey's test).

To evaluate if the stress phytohormone ABA played a role in the drought response of *MaSp1*‐tobacco, ABA content in the shoots was evaluated for WT, *MaSp1*_*nuc*_ (#1 and #3), and *MaSp1*_*pla*_ (#2–2‐8 and #2–2‐9) plants under drought stress (14‐day‐old seedlings in soil – water withheld for 22 days without recovery) (Figure 5a) and well‐watered conditions (37‐day old seedlings in soil) (Figure 5b). ABA content in *MaSp1*‐tobacco under drought remained similar to WT (Figure 5a), but was significantly higher than the WT in well‐watered conditions (Figure 5b). This corresponded to an increased expression of ABA biosynthesis genes – 9‐*cis*‐epoxycarotenoid dioxygenase (*NCED3*) and xanthoxin dehydrogenase (*ABA2*) (Figure 5c top panel) in well‐watered *MaSp1*‐tobacco. We also observed an increase in the expression of ABA signaling genes – protein phosphatase 2C (*PP2C*) and mitogen‐activated protein kinase kinase (*MAPKK9*) (Figure 5c middle panel), and the ABA catabolic gene cytochrome P450 (*CYP707A1*) (Figure 5c bottom panel) in these plants. An increase in endogenous ABA in well‐watered *MaSp1*‐tobacco upregulated the key players of the ABA signaling pathway, including *CYP707A1*, which is essential for ABA homeostasis, pivotal to the adaptation of plants to abiotic stress (Truong et al., 2021). The infrared images of leaves of well‐watered plants revealed a significantly higher leaf temperature in *MaSp1*‐tobacco than the WT (Figure 5d,e) indicating ABA‐induced stomatal closure in these lines in well‐watered conditions. This was also consistent with the significantly lower water loss from detached leaves of *MaSp1*‐tobacco as evaluated using a rapid dehydration detached‐leaf assay which measures the short‐term water loss avoidance response in plants (Figure 5f). In addition, significantly higher TAS was observed in well‐watered *MaSp1*‐tobacco compared with the WT (Figure 5g). Thus, a moderate increase in ABA content in well‐watered *MaSp1*‐tobacco led to stomatal closure and reduced water loss from leaves, along with the modulation of ABA signaling and catabolism, and an increase in TAS. These physiological changes are similar to those observed during ABA priming.

### ABA priming leads to drought tolerance in *MaSp1* ‐tobacco

The higher ABA content and consistently higher TAS in *MaSp1*‐tobacco under normal growth conditions suggested the possibility of ABA priming as a key mechanism for *MaSp1*‐induced drought tolerance. ABA priming results in faster or enhanced modulation in the expression of stress‐responsive genes upon exposure to stress (Liu, Quan, & Bartels, 2022). Hence, we examined the DEGs in *MaSp1*‐tobacco under drought with more than twofold differences in expression compared with the WT that were responsive to ABA or stress and had previously reported roles in stress signaling and tolerance (Table S2). To select these genes, we performed manual cross‐referencing of our results against the literature.

**Figure 6:**
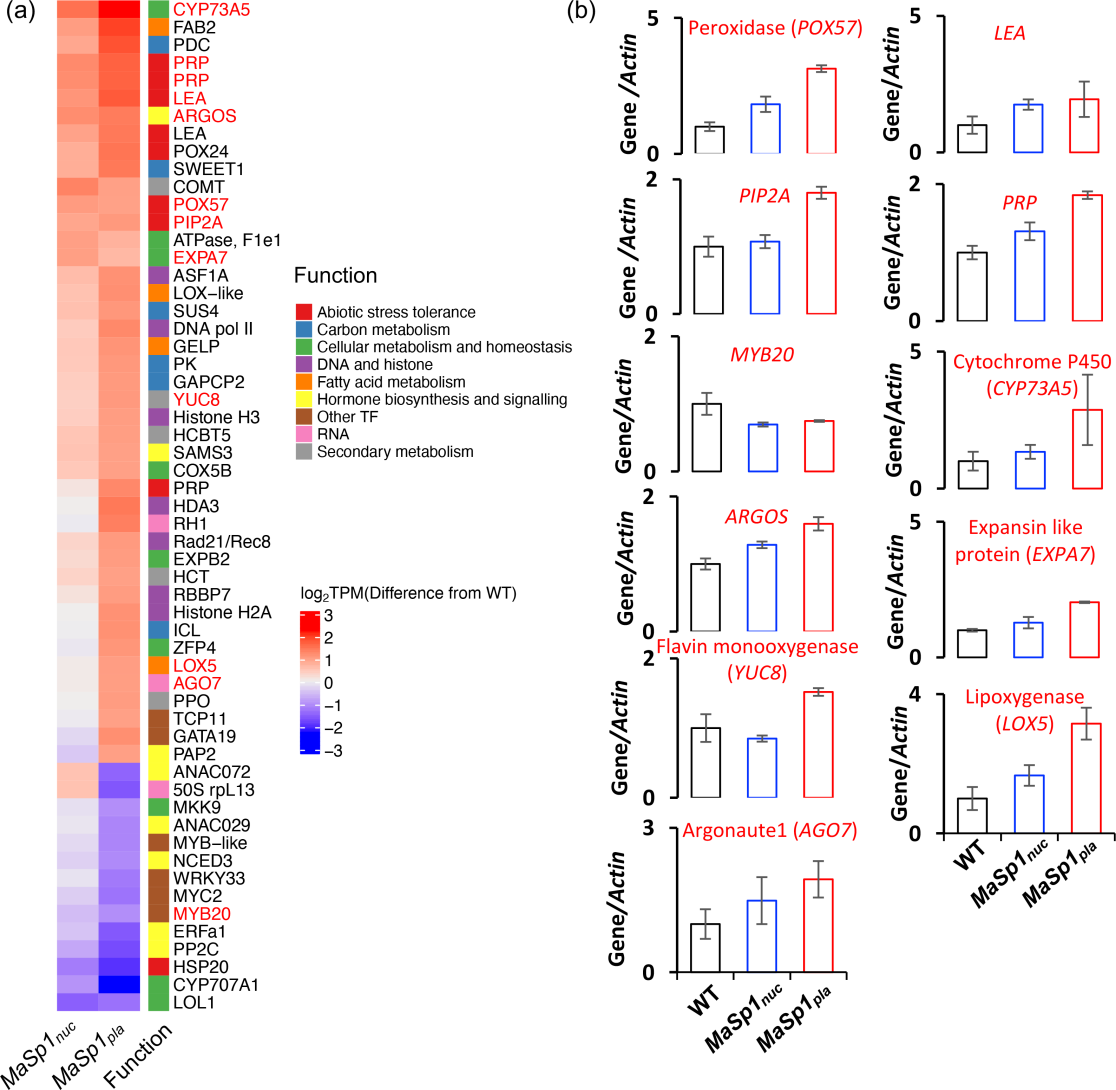
Transcriptional regulation of genes in drought‐stressed (ψ_w_ − 0.63 MPa) *MaSp1*‐tobacco with previously reported roles in stress tolerance. (a) Heatmap of DEGs (more than twofold difference in expression relative to the WT) which are responsive to ABA or stress and previously implicated in stress tolerance in tobacco or other plant species as shown in Table S2, and (b) RT‐qPCR confirmation of few key players in drought tolerance (shown in red in (a)), expressed as relative abundance to *Actin* in shoots from *n* = 15 plants, where the gene/*Actin* ratio for WT was set to 1. Data represent means ± SEM.

We identified several genes among the DEGs, related to abiotic and biotic stress tolerance (Table S2), carbon metabolism, cellular metabolism, cellular homeostasis, RNA, DNA and histone‐related functions, hormone biosynthesis and signaling, and secondary metabolism (Figure 6a). Many of these genes were also reported to be transcriptionally responsive to ABA and various abiotic stresses in tobacco or other plant species (Table S2). The extent of upregulation or downregulation of most of these genes was higher in *MaSp1*_*pla*_ than in *MaSp1*_*nuc*_ (Figure 6a; Table S2). However, certain genes, including those encoding an auxin‐inducible, phytochrome‐associated protein *PAP2*, the ABA‐mediated drought tolerance transcription factor *ANAC072*, and ribosomal protein L13, were oppositely regulated in *MaSp1*_*nuc*_ and *MaSp1*_*pla*_ (Figure 6a; Table S2).

To validate the RNA‐seq data, we performed RT‐qPCR on key genes with previously reported roles in drought tolerance or transcriptional response to ABA or drought stress (Figure 6b; Table S2). The plant peroxidase gene *POX57* which is involved in mitigating oxidative stress (Pandey et al., 2017; Yoshida et al., 2003), the stress‐related *LEA* (Chen et al., 2019) and *PRP* genes (Xu, Zheng, et al., 2018), and *EXPANSINA7* (*EXPA7*) (Li et al., 2011), which help maintain cellular stability and homeostasis under drought stress, were upregulated in *MaSp1*‐tobacco, with a stronger expression in *MaSp*_*pla*_ compared with *MaSp1*_*nuc*_. Additionally, both the aquaporin gene *PIP2A* which helps maintain cellular water homeostasis (Zargar et al., 2017) and *YUC8* which is involved in auxin biosynthesis and regulating drought tolerance (Cao et al., 2019) were upregulated in *MaSp1*_*pla*_. *MYB20*, a negative regulator of drought tolerance (Gao et al., 2014), was downregulated in *MaSp1*‐tobacco. Other upregulated genes in *MaSp1*‐tobacco (more so in *MaSp1*_*pla*_ than *MaSp1*_*nuc*_) included genes encoding *Cytochrome P450 73A5* [*CYP73A5*], a precursor of phenylpropanoid biosynthesis (Bell‐Lelong et al., 1997; Wang et al., 2017; Xu et al., 2022), *ARGOS*, a negative regulator of ethylene signaling (Kuluev et al., 2019; Shi et al., 2015), *ARGONAUTE1*, a silencing effector that mediates microRNA‐mediated drought tolerance (Westwood et al., 2013), and *LOX5*, a lipoxygenase involved in the oxygenation of linoleic and linolenic acid (Feussner & Wasternack, 2002; Hou et al., 2015; Lim et al., 2015; Xing et al., 2020); all of which play roles in drought tolerance (Figure 6b; Table S2).

We also evaluated the transcriptional expression of these genes (Figure 6b) in unstressed plants cultivated in MS medium at the same growth stage (Figure S11) and observed higher gene expression in *MaSp1*‐tobacco relative to WT for *POX57*, *PIP2A*, *LEA*, *PRP*, *CYP73A5*, and *EXPA7* (Figure S11, left panel). However, no differences between *MaSp1*‐tobacco and the WT were observed for *MYB20*, *ARGOS*, *YUC8*, and *AGO7* (Figure S11, right panel). These results suggest that the moderately higher ABA levels in MaSp1‐tobacco may have a secondary effect on drought tolerance, conceivably due to ABA priming, which modulates the transcription of genes involved in drought tolerance even in unstressed conditions.

In conclusion, a higher endogenous ABA content and the primary effects of this on stomatal closure, lower water loss, higher antioxidant status, and activation of stress response due to ABA signaling and homeostasis were identified as the key mechanisms in *MaSp1*‐induced drought tolerance. However, multiple mechanisms with cumulative roles on *MaSp1*‐induced drought tolerance, such as those involving epigenetics (RNA, DNA, and histone modifications), transcription (transcription factor genes *MYB20, ANAC072*), phytohormones (*YUC8, ARGOS*), transporter genes (*NRT, PIP2A, SWEET1*), various proteins (*POX57, EXPA7*, *LEA*, *PRP*, *LOX5*, *CYP73A5*, *L13*), and metabolites (phenylpropanoids, alkaloids, flavonoids, selenocompounds) were also observed. Thus, we also speculate that ABA priming caused by higher endogenous ABA content induces overall adjustments in multiple drought tolerance mechanisms and hence confers drought tolerance to these plants.

## DISCUSSION

Here, we produced spider silk protein (MaSp1 with six‐repeat domains) in tobacco by transforming its nuclear and plastid genomes and evaluated the role of this protein in determining the mechanical properties of plant material, particularly leaves. In the field of biomaterials science, the use of synthetic fillers in biological matrices is a common approach for enhancing the inherent properties of biocomposites (Di Giacomo et al., 2015; Haneef et al., 2017; Jones et al., 2018; Valentini, Bittolo Bon, et al., 2016; Valentini, Bon, et al., 2016). We adopted this approach by introducing *MaSp1* in the nuclear and plastid genomes of tobacco to produce spider silk protein as a natural filler in the cells and tissues of tobacco plants.

Given the structural roles of glycine‐rich proteins in plants, one of our goals was to determine whether the production of glycine‐rich repeat domains of spider silk protein in tobacco would improve its endogenous structural traits. Although we did not detect significant improvement in the tensile properties of leaf tissue in the *MaSp1*‐tobacco lines (Figure 2), we believe that targeting this protein to other locations in the cell may enhance plant mechanical properties. Specific signal peptides that would help localize GRPs to the cell wall, vascular tissues, or extracellular spaces can be engineered at the N‐ or C‐terminus of glycine‐rich MaSp1 to ensure its targeting to the respective structural components of the plant cell.

Tobacco leaves are composed of upper and lower epidermal cell layers, a mesophyll layer composed of parenchymatous cells, and vascular bundles. Despite their weak mechanical properties, parenchyma cells can contribute to the overall mechanical efficiency of plants (Gibson, 2012). The Young's modulus and tensile strength of leaves at both 85% and 15% moisture content observed in this study were consistent with previous results for parenchyma cells (Gibson, 2012) and tobacco leaves (Ganeriwala, 1990; Henry et al., 2000). The tensile properties, tensile strength (Figure 2c), strain at break (Figure 2d), and toughness (Figure 2f) tended to decrease with decreasing leaf moisture, but Young's modulus (an indicator of stiffness) increased 2.5‐fold (Figure 2e), which is consistent with previous findings (Henry et al., 2000). Hence, the simple procedure employed in this study can be used for tensile testing of leaves and/or leaf‐based biomaterials, representing an improvement over the elaborate procedures demonstrated in earlier studies (Ganeriwala, 1990; Henry et al., 2000; Sahaf & Sharon, 2016). Furthermore, at 15% leaf moisture, the toughness of *MaSp1*‐tobacco tended to be higher than that of the WT and *GFP* controls (Figure 2f). Leaf lamina toughness depends on the volume fraction of the tissue occupied by the cell wall (Choong, 1996), suggesting that either the cell density or cell wall thickness was higher in the leaves of *MaSp1*‐tobacco relative to the WT. Although additional research is needed to confirm this phenomenon, our findings point to the thickening of the cell wall due to the production of the glycine‐rich protein MaSp1 as a stress tolerance mechanism (Le Gall et al., 2015; McCahill & Hazen, 2019).

We expressed a spider silk protein (MaSp1) in plant plastids for the first time and observed that the accumulation of this protein in plastids (*MaSp1*_*pla*_ #2–2) resulted in its dimerization, as shown by immunoblotting of total soluble proteins from transplastomic leaves (Figure 1i). This dimerization may be due to a higher abundance of hydrophobic monomers, as it was also observed in the His‐trap purified fractions of *MaSp1*_*nuc*_ (Figure S2a). However, the LC–MS/MS data from gel‐excised bands at approximately 25 kDa (size of the monomer) in *MaSp1*_*pla*_ #2–2 (Figure S2a) showed a peptide coverage of 67% (Dataset S1 and Figure S2d), which revealed the cleavage of the His‐tag in MaSp1 at the 45th amino acid position of the translated sequence (Figure S2e,f), possibly by a stromal processing peptidase. This hindered the purification and accurate quantification of the accumulation of MaSp1 in the transplastomic lines. Thus, despite an expected increase in MaSp1 in *MaSp1*_*pla*_, it showed similar protein yields as *MaSp1*_*nuc*_ (Figure S3b,c).

*MaSp1*‐tobacco displayed improved growth under drought conditions (Figure 3b,c,e), which coincided with the upregulation of genes involved in cellular catabolism (GO term: Catabolism) (Figure 4b). However, we observed the downregulation of genes involved in photosynthesis (Figure 4b) in drought‐stressed *MaSp1*‐tobacco. We believe that this inconsistency reflects the compromised photosynthesis and growth due to MaSp1 accumulation itself rather than the effects of drought, as demonstrated by the lower DW of *MaSp1*‐tobacco under well‐watered conditions at 52 DAS (Figure S4a).

We also observed that *MaSp1*_*pla*_ exhibited better drought response and recovery compared with *MaSp1*_*nuc*_ plants under mild drought stress and drought treatment in soil (Figure 3c,e), which were consistent with the TAS values of these lines (Figure 4c). Genes in KEGG terms involving the biosynthesis of additional antioxidant secondary metabolites were upregulated only in *MaSp1*_*pla*_ but not *MaSp1*_*nuc*_ (Figure S10b), corresponding to the higher TAS values of *MaSp1*_*pla*_ (Figure 4c). Additionally, a higher MaSp1 content in *MaSp1*_*pla*_ might also contribute to the differences observed between the drought responses and TAS of *MaSp1*_*pla*_ and *MaSp1*_*nuc*_. However, precise quantification of protein content was challenging due to the cleavage of the His‐tag as previously mentioned. Furthermore, various genes were upregulated in *MaSp1*_*pla*_ but not in *MaSp1*_*nuc*_ under drought stress, including genes involved in DNA synthesis, replication, and repair (GO: DNA metabolic process); RNA synthesis, processing and modification (GO: RNA metabolic process); protein methylation and methyltransferase activity (GO: methyltransferase activity); and ribonucleoprotein complex, ribosome biogenesis and assembly, and rRNA binding and translation (GO: RNA binding and ribosome) (Figure 4b). This differential upregulation of genes involved in DNA and RNA modifications points to the possible influence of epigenetic mechanisms in drought tolerance (Lämke & Bäurle, 2017; Sun et al., 2021) in *MaSp1*_*pla*_. These results also indicate possible differences in the drought tolerance mechanisms between *MaSp1*_*pla*_ and *MaSp1*_*nuc*_; the opposite expression patterns of genes encoding PAP2, ANAC072, and ribosomal protein L13 between *MaSp1*‐tobacco genotypes support this notion (Figure 6a). The possible contribution of epigenetic mechanisms to drought tolerance in *MaSp1*_*pla*_ also points to differences in the duration of stress memory (Lämke & Bäurle, 2017) between *MaSp1*_*nuc*_ and *MaSp1*_*pla*_. These differences could be due to the different localizations of MaSp1, a concept that requires further study.

Previous studies have demonstrated the role of GRPs in imparting drought stress tolerance (Ciuzan et al., 2015; Yang et al., 2014), but the underlying mechanism is unknown. Most reports attribute this effect to the RNA and DNA binding ability of the proteins, which stabilize various transcripts under stress conditions (Fusaro et al., 2007; Czolpinska and Rurek., 2018; Xu et al., 2022; Shim et al., 2021). Here, preliminary sequence analysis of MaSp1 using PredictProtein (Bernhofer et al., 2021; Qiu et al., 2020) revealed a DNA binding ability for this protein, with a high‐reliability index (Figure S12a). The repetitive RGG motifs in the glycine‐rich MaSp1 repeat domain (6×) (Figure S1a) may also facilitate RNA binding and contribute to key cellular programs such as transcription, splicing, mRNA export, and post‐translational modifications, as observed in previous studies (Gao et al., 2019; Rajyaguru & Parker, 2012; Wong et al., 2010).

Furthermore, the glycine‐rich regions of MaSp1 are intrinsically disordered and show a tendency for liquid–liquid phase separation (LLPS) (Figure S12b), as demonstrated by a prediction algorithm ParSe (Paiz et al., 2021). These observations raise a possibility that MaSp1 undergoes phase separation and binds to DNA and RNA. However, exactly how ABA levels are regulated by MaSp1 production in tobacco, and whether the predicted phase separation of MaSp1 is linked to ABA biosynthesis remain elusive and require further investigation.

In this study, we demonstrated that expressing a gene encoding a spider silk protein from the nucleus and plastids of tobacco improved drought response and recovery, with *MaSp1*_*pla*_ showing increased drought resilience compared with *MaSp1*_*nuc*_. However, no significant improvements in the tensile properties of leaves were observed. Nonetheless, our findings lay the foundation for the use of agricultural biomass wastes from drought‐resilient crops as a source of material production with structural applications. However, this may face regulatory challenges and public opposition. Targeting *MaSp1*‐like sequences for drought tolerance in non‐food crops for industrial uses, such as biofuel production could encounter fewer regulatory hurdles. Several countries have adopted genome‐edited products for food, feed, and biomass but their regulatory policies are inconsistent. Moving forward, clear communication and aligned global policies are essential for the widespread adoption of genome‐edited crops for a sustainable future.

## MATERIALS AND METHODS

### Plant material and growth conditions

Tobacco (*Nicotiana tabacum* cv. Petit Havana SR1) seeds were surface sterilized using 75% (v/v) ethanol for 2 min, followed by 10% (v/v) commercial bleach (Kao, Japan) for 15 min, and washed with sterile MilliQ water 5 times. The disinfected seeds were sown on soil (potting soil mix [Promix, Rivière‐du‐Loup, Canada] and vermiculite in a 2:1 ratio) and placed in a growth chamber at 25°C, 14‐h light/10‐h dark photoperiod, 75% relative humidity, and 200 μmol photons m^−2^ s^−1^. Nutrient solution (Hyponex, Japan) at an N:P:K ratio of 6:10:5 diluted 1000‐fold was provided each week.

To obtain sterile leaves for *Agrobacterium*‐mediated transformation, particle bombardment, and drought experiments, surface‐sterilized seeds were sown on Murashige and Skoog (MS) salt and vitamins (Merck, USA) medium supplemented with 3% (w/v) sucrose and solidified with 0.25% (w/v) Phytagel (hereafter referred to as MS medium) and placed in a growth chamber at 25°C, 14‐h light/10‐h dark photoperiod, 60% relative humidity, and 100 μmol photons m^−2^ s^−1^. Unless otherwise specified, these growth chamber settings were maintained for all seedling experiments.

### Plasmid DNA construction

#### Nuclear transformation

pET30‐a‐MaSp1 harboring the *MaSp1* repeat domain (6×) from the spider *T. clavipes*, with His‐tag, S‐tag, thrombin, and enterokinase sequences (Prince et al., 1995) (denoted as *MaSp1*) was used as the template for the insertion of a *Sac*I recognition site at the 3′ end of *MaSp1* via point mutation of an existing *Bsp1286*I recognition site from GAGCAC to GAGCTC using site‐directed mutagenesis PCR (LATaq DNA Polymerase, Takara Bio Inc., Japan) with primers F1 and R1 (Table S3). The PCR product was treated with *Dpn*I (Takara Bio Inc., Japan) to eliminate the parent template. The pBI121 vector and the *Dpn*I‐digested PCR product were double‐digested with the restriction enzymes *Xba*I and *Sac*I (Takara Bio Inc., Japan). The digested products were gel‐extracted (QIAquick Gel Extraction kit, QIAGEN, Germany) and ligated at a 1:3 (vector: insert) molar ratio using Ligation high (Toyobo, Japan). The ligated product harbored *MaSp1* under the control of the CaMV *35S* promoter, a *Nos* terminator, and the *NPTII* selection marker (which confers resistance to kanamycin) to generate pBI121_m_ (Figure S1b).

#### Plastid transformation

*Nde*I‐digested pPRV112AG harboring an *aadA* and e*GFP* expression cassette was purified (QIAquick PCR Purification kit, QIAGEN, Germany) and used as a template for PCR amplification of the region flanking e*GFP* using KOD‐Plus DNA Polymerase (Toyobo, Japan) and primers F2 and R2 (Table S3), resulting in the amplification of an open linear vector. *MaSp1* was PCR amplified using primers F3 and R3 (Table S3) and LATaq DNA Polymerase (Takara Bio Inc., Japan). Both the PCR‐amplified open vector and the *MaSp1* amplicon were subjected to gel extraction (QIAquick Gel Extraction kit, QIAGEN, Germany), and the latter was introduced into the open vector using an In‐fusion HD Cloning kit (Takara Bio Inc., Japan) according to the manufacturer's instructions. The resulting product harbored *MaSp1* under the control of the *psbA* promoter, an *rps16* terminator (*P*_*psbA*_:*MaSp1*‐T_*rps16*_), the *aadA* selection marker within the *16S* rDNA and *rps7/12* homology region, and an ampicillin selection marker in the pBluescriptSK+ background. This vector is referred to as pPRV112A_m_ hereafter (Figure S1c).

The in‐fusion product (2.5 μL) and the ligated product (4 μL) were independently added to 50 μL of chemically competent *E. coli* DH5α cells, which were transformed by heat‐shock transformation as previously described (Froger & Hall, 2007). A 100‐μL aliquot of cells was plated on Luria‐Bertani (LB) agar (BD Difco, US) containing 50 mg/mL of kanamycin or 100 mg/mL of ampicillin for pBI121_m_ and pPRV112A_m_, respectively, and incubated at 37°C overnight in the dark. Colonies resistant to the corresponding antibiotics were selected to confirm successful transformation using colony PCR (GoTaq Green Master Mix, Promega Corporation, US) and the primers F4 and R4 (Table S3). After sequence verification by DNA sequencing (BigDye® Terminator v3.1, Life Technologies, US), positive colonies were cultured in LB broth (BD Difco, US) containing 50 mg/mL of kanamycin or 100 mg/mL of ampicillin for pBI121_m_ and pPRV112A_m_, respectively, at 37°C for 10 h. Plasmid DNA (pBI121_m_ and pPRV112A_m_) was isolated from the cells using a QIAprep Spin Miniprep kit (QIAGEN, Germany).

### Agrobacterium‐mediated transformation

Electroporation of *Agrobacterium* strain GV3101 or LBA4404 with pBI121.MaSp1 or pBI121.GFP plasmid DNA, respectively, was performed as recommended by the manufacturer (MicroPulser electroporator, Bio‐Rad, CA, US). The cells were plated on YM plates (10 g/L mannitol, 0.4 g/L yeast extract, 0.1 mg/L K_2_HPO_4_, 0.4 mg/L KH_2_PO_4_, 0.1 mg/L NaCl, 0.2 mg/L MgSO_4_.7H_2_O, 15 g/L agar, pH 6.8) containing 50 mg/L kanamycin and grown in the dark at 30°C for 2 days. Colonies carrying MaSp1 were confirmed by colony PCR (GoTaq Green Master Mix, Promega Corporation, US) using the primers F4 and R4 (Table S3). The confirmed colonies were inoculated in YM broth containing 50 mg/L kanamycin and grown in the dark at 30°C for 2 days. Cocultivation and regeneration were performed as previously described (Gallois & Marinho, 1995) with the following modifications. The Agrobacterium cultures were diluted to OD_600_ = 0.6 with YM broth containing 100 μM acetosyringone and incubated with leaf disks for 5 min. The leaf disks were incubated on a cocultivation medium in the dark for 1 day, followed by cultivation under a 16‐h light/8‐h dark photoperiod for 2 days. The regeneration medium included MS salt and vitamins (Merck, USA) and contained 300 mg/L of cefotaxime, 50 mg/L of kanamycin, 1 mg/L of 6‐benzylaminopurine, and 0.1 mg/L of 1‐naphthaleneacetic acid.

### Plastid transformation via particle bombardment

Particle bombardment of *N. tabacum* leaves with pPRV112A_m_ and pPRV112AG (Figure S1c) was independently carried out as previously described (Okuzaki & Tabei, 2012) with 2.5 μg plasmid DNA per plate using a PDS‐1000/He biolistic particle delivery system (Bio‐Rad, US).

### Genotyping and gene expression analysis

Genomic DNA from regenerated shoots was extracted in an extraction buffer (1 M KCl, 100 mM Tris–HCl (pH 8.0), and 10 mM EDTA) after incubation at room temperature for 15 min with vortexing. The mixture was centrifuged at 13000 ***g*** for 10 min at room temperature and the supernatant was mixed with equal volumes of isopropanol. The mixture was centrifuged again at 13000 ***g*** for 10 min at 4°C, and the pellet was washed with 80% ethanol. The washed pellet was air‐dried for 10 min and resuspended in 30 μL of sterile milliQ. Regenerated shoots were screened for the presence of *MaSp1* in the nuclear genome and the plastome by GXL DNA polymerase (Takara Bio Inc., Japan) using 1 μL of the template DNA and the primers F4 and R4, and F5 and R5, respectively (Table S3).

Southern blotting was performed with 10 μg of genomic DNA from T1 (#2–2) and T2 progeny (#2–2‐8 and #2–2‐9) of *MaSp1*_*pla*_#2–2 which were separated on a 1% Agarose gel following overnight restriction digestion with *SacII* (Takara Bio Inc., Japan). The DNA fragments were transferred to a positively charged nylon membrane (Amersham Hybond‐N+, Cytiva, Japan). Hybridization with a DNA probe labeled with alkaline phosphatase (Amersham AlkPhos Direct labeling modules, Cytiva, Japan), high stringency washes, and detection (CDP‐Star Detection Reagent, Cytiva, Japan) were performed according to manufacturer's instructions. The DNA probe was designed to target the *trnV*–*16SrDNA* region using specific primers and GXL DNA polymerase (Takara Bio Inc., Japan) (Table S3).

Total soluble protein was extracted from approximately 100 mg frozen leaf tissue using Rluc‐Lysis Buffer (Promega Corporation, USA). The sample was centrifuged at 10000 ***g*** for 20 min, and the supernatant was collected. Protein concentrations were measured using Bradford reagent (XL‐Bradford, Apro Science, Japan). Total soluble proteins were resolved using SDS‐PAGE (4%–15% Mini‐Protean TGX precast gel, Bio‐Rad, US) under reducing conditions. Seven and 15 μg of total soluble protein were loaded in each well for the transgenic and transplastomic lines, respectively. Immunoblotting was performed as previously described (Foong et al., 2020) with a mouse monoclonal anti‐6 × His primary antibody (Agrisera, Sweden, AS11‐1771, 1:1000) and a goat‐anti‐mouse IgG HRP‐conjugated secondary antibody (Abcam, United Kingdom, ab 6789, 1:10000).

### Confirmation of recombinant MaSp1 by LC–MS/MS

Total soluble proteins from *MaSp1*_*nuc*_ and *MaSp1*_*pla*_ lines were His‐trap purified as previously described (Foong et al., 2020) and resolved on SDS‐PAGE gels (4%–20% Mini‐PROTEAN TGX, Bio‐Rad, US). Bands corresponding to 25 kDa and 50 kDa for MaSp1_pla_ and 25 kDa for MaSp1_nuc_ were excised, destained, and digested with TPCK‐treated trypsin (Worthington Biochemical Corporation, US). The resulting peptides were separated on a reversed‐phase nanospray column (NTCC‐360/75–3‐105, NIKKYO Technos, Japan) and applied to a Q Exactive Hybrid Quadrupole‐Orbitrap mass spectrometer (Thermo Fisher Scientific, US). MS and MS/MS data were obtained using the TOP10 method. The data were processed using Proteome Discoverer 2.4 (Thermo Fisher Scientific, US) with integrated Mascot (version 2.7, Matrix Science). The MS/MS data were searched against the NCBIprot database (Taxonomy: Other green plants) and an in‐house database including MaSp1 using the following parameters: enzyme = trypsin; maximum missed cleavages = 3; variable modifications = Acetyl (Protein N‐term), Gln‐ > pyro‐Glu (N‐term Q), Oxidation (M), Propionamide (C); product mass tolerance = ± 15 ppm; product mass tolerance = ± 30 milli mass unit; instrument type = ESI‐TRAP.

### Tensile test

The first or second fully expanded leaf (from the top) was excised from a plant and used for tensile tests. A die with a 2‐cm gauge length and 2 mm in thickness was placed between two lateral veins in the center of the abaxial side of the leaf (Figure S6a), and leaf cut‐outs were obtained using a punching setup (Figure S6b). Fresh and moisture‐conditioned leaf cut‐outs were fastened onto the clamps of a 1 N load cell of the tensile tester (EZ‐LX, Shimadzu Corporation, Japan) (Figure S6c), and measurements were carried out with a stroke speed of 1 mm/min. Twelve to twenty leaf cut‐outs from four individual plants were obtained and subjected to tensile testing after moisture conditioning.

### Leaf moisture conditioning

Leaf cut‐outs were obtained from the first or second fully expanded leaves (~10 cm long) from four individual plants per genotype. The leaf cut‐outs were incubated in a chamber containing saturated K_2_SO_4_ solution at room temperature to maintain the relative humidity at 75% for 70–80 h until the leaf moisture was reduced to below 20%. The % leaf moisture after conditioning (*LmAC*) was obtained using the following equation:

$$\text{LmAC}\left(\%\right)=\frac{\mathrm{TLm}\left(\%\right)}{100}\times \frac{\text{LmAC}\left(g\right)}{\mathrm{TLm}\left(g\right)}$$

where *TLm*(%) and *TLm*(g) are %(w/w) and grams of total leaf moisture, respectively, and *LmAC(g)* is grams of leaf moisture after conditioning. The leaf cut‐outs were weighed before and after placing them into the moisture chamber and after completely drying them at 70°C for 2 weeks.

### Plant growth characteristics

A micro‐caliper was used to measure the thickness of leaves used for the tensile test. Leaf thickness is expressed as the average of three to four values at different positions on the leaf. The leaf areas used for the tensile tests were obtained using ImageJ 1.52q, and plant height was measured from the base of the stem to the tip of the newest leaf. To obtain the dry weight (DW) of plants used for tensile testing, the leaves and stem of each plant were separately sampled and dried at 60°C for 2 weeks before weighing. The growth analysis of *MaSp1*‐tobacco was conducted by evaluating the single‐shoot DW of genotypes grown in soil at 8 DAS (*n* = 3–6 sets, 7 plants each set), 15 DAS (*n* = 3–6 sets, 7 plants each set), 22 DAS (*n* = 5–10 sets, 3 plants each set), 37 DAS (*n* = 7–10 sets, 3 plants each set) and 52 DAS (*n* = 5 plants) after drying at 60°C for 2 weeks.

### Drought stress studies

WT seedlings and seedlings of two independent *MaSp1*_*nuc*_ lines (#1 and #3), T2 progeny of *MaSp1*_*pla*_ #2–2 (#2–2‐8, and #2–2‐9), and their corresponding vector controls (*GFP*_*nuc*_, and *GFP*_*pla*_) were cultivated as described above. The DWs of 30 seedlings per line were obtained 13 days after sowing (DAS), that is, before drought induction. Thirty 13‐day‐old seedlings were transferred to PEG‐infused MS medium with a water potential (ψ_w_) of −1.42 MPa (Verslues et al., 2006) for drought stress treatment. At 7 days after drought induction (DAD), the seedlings were rescued by transferring them to MS medium, and at 8 days after recovery (DAR), the DW of the rescued seedlings was measured. Seedlings with a green emerging leaf were considered to be rescued. To measure the % decrease in single seedling DW due to drought, unstressed seedlings at 22 DAS and rescued seedlings at 2 DAR were sampled and their DWs were obtained.

For the mild drought stress experiment, *MaSp1*_*nuc*_ #1, *MaSp1*_*nuc*_ #3, *MaSp1*_*pla*_ #2–2‐8, and *MaSp1*_*pla*_ #2–2‐9 seedlings were grown alongside WT seedlings on 1% (w/v) agar for 14 days, transferred to PEG‐containing medium (ψ_w_ − 0.63 MPa (Verslues et al., 2006)), incubated for 9 days, and their DWs obtained at 2 DAR. The increase in DW under drought stress was calculated relative to the DW of the seedling before drought induction.

For drought studies in soil, 16‐day‐old seedlings grown on 1% (w/v) agar (Merck, US) were transferred to the soil under well‐watered conditions. After 3 days in soil, watering was withheld for 16 days until the leaves started to wilt, which was considered to be the onset of drought at which stage water was provided for recovery. Shoot DWs were recorded at 2 DAR and 0 DAD to obtain the relative increase in DW under water withholding treatment.

### Measuring ABA levels

WT, *MaSp1*_*nuc*_ (#1 and #3), and *MaSp1*_*pla*_ (#2–2‐8 and #2–2‐9) plants were grown on soil under conditions described above (in “Plant material and growth conditions”) with the same amount of soil and water in each pot. Shoots of 37‐day‐old plants for well‐watered treatment and 36‐day‐old plants (14‐day‐old seedlings – water withheld for 22 days) for drought stress treatment were immediately frozen in liquid N_2_, lyophilized, and ground with beads in a mixer mill (MM 400; Retsch, Germany). The samples were homogenized with 80% (v/v) acetonitrile containing d6‐ABA (Icon Isotopes, USA). After 5 min of incubation, the samples were centrifuged to remove debris, and the pellets were rewashed. The supernatant was concentrated using a SpeedVac (Thermo Fisher Scientific, USA). The samples were filtered through a membrane filter by centrifugation, and the solvent was subjected to LC‐ESI‐MS/MS analysis on a Triple Quad 5500 system (AB Sciex, Canada) coupled to a NexeraX2 HPLC instrument (Shimadzu, Japan) equipped with a TSKgel ODS‐120H column (2.0 mm ID × 5 cm, 1.9 μm; Tosoh Bioscience, Japan). Analytical conditions for LC and ESI‐MS/MS were essentially as described previously (Mega et al., 2019).

### Infrared imaging analysis and transpirational water loss assay

Plants were grown in soil pots for 30 days at 23 ± 2°C. Leaf surface temperature was monitored with an R300SR‐S Infrared imaging camera (Nippon Avionics, Japan). Temperatures on the leaf surface were determined using InfReC Analyzer NS9500 software (Nippon Avionics). Photos of plants were taken with an EM‐5 Mark II digital camera (Olympus, Japan). For the transpirational water loss assay, plants were grown in soil pots for 30 days at 23 ± 2°C. The aerial parts were detached from roots, and their fresh weights were measured at the beginning, 1 and 2 h, respectively.

### Total antioxidant status (TAS) assay and RT‐qPCR

Fifteen‐day‐old WT, *MaSp1*_*nuc*_ (#1 and #3), and *MaSp1*_*pla*_ (#2–2‐8 and #2–2‐9) seedlings were grown in PEG‐containing medium (ψ_w_ − 0.63 MPa (Verslues et al., 2006)) for 9 days, and 15 or 20 seedlings per genotype were ground to a powder in liquid N_2_ and subjected to RT‐qPCR and TAS assays, respectively. Sixteen‐day‐old WT, *MaSp1*_*nuc*_ (#1 and #3), and *MaSp1*_*pla*_ (#2–2‐8 and #2–2‐9) seedlings grown on MS medium (15 seedlings) were also subjected to RT‐qPCR. RNA extraction was performed using RNeasy Plant Mini Kit (QIAGEN, Germany); 500 ng of RNA was used for first‐strand cDNA synthesis using ReverTraAce qPCR RT Master Mix with gDNA Remover (Toyobo, Japan). One microliter of fivefold diluted cDNA was used for qPCR using SYBR‐green Real‐time PCR Master Mix Plus (Toyobo, Japan). Primers used for qPCR are listed in Table S3. The comparative 2^–ΔΔCT^ method was used to evaluate the differential expression of genes relative to *Actin*; the relative expression level of each gene/*Actin* for WT was set to 1.

TAS was expressed as mM Trolox equivalent/(kg FW or kg DW) for WT, *MaSp1*_*nuc*_, and *MaSp1*_*pla*_ plants under water‐stressed (ψ_w_ − 0.63 MPa (Verslues et al., 2006)) and non‐stressed conditions using a total Antioxidant Status kit (Elab Science, USA) according to the manufacturer's instructions.

### RNA sequencing

Fourteen‐day‐old WT, *MaSp1*_*nuc*_ (#1 and #3), and *MaSp1*_*pla*_ (#2–2‐8_p_ and #2–2‐9) seedlings were grown in PEG‐containing medium (ψ_w_ − 0.63 MPa (Verslues et al., 2006)) for 11 days, and 8 seedlings per genotype were ground to a powder in liquid N_2_ for RNA extraction using an RNeasy Plant Mini Kit (QIAGEN, Germany). RNA samples extracted from eight seedlings of the corresponding genotype were pooled for RNA sequencing and subjected to DNase treatment. After confirming the quality and integrity of the RNA, sequencing libraries were prepared (TruSeq Stranded mRNA Library prep, Illumina Inc., US) and subjected to 100 bp paired‐end sequencing (NovaSeq 6000, Illumina Inc., US). The quality of the RNA used for library preparation and paired‐end sequencing was evaluated by Macrogen Japan.

All reads were trimmed using fastp version 0.20.1 (Chen et al., 2018) using the following parameters: ‐‐detect_adapter_for_pe, −3, −l 30, −q 30. The trimmed reads were mapped onto the reference sequences of the Nitab‐v4.5_genome_Chr_Edwards2017 (Edwards et al., 2017), rRNA sequences of *Nicotiana tabacum* collected in the Rfam database version 14.8 (Kalvari et al., 2021), and mitochondrial and chloroplast genomes (GenBank accession numbers NC_006581.1 and NC_001879.2, respectively) using STAR version 2.7.9a (Dobin et al., 2013) and RSEM version 1.3.3 (Li & Deway, 2011) with default parameters.

The expression level of each gene was normalized to transcripts per kilobase million (TPM) (Li & Deway, 2011) using the raw count data mapped to Nitab‐v4.5_genome_Chr_Edwards2017. Log2(TPM) was calculated as Log_2_(TPM + 1); 11.7, 11.9, and 12.2 million reads per sample were used to calculate the TPM of *MaSp1*_*nuc*_, *MaSp1*_*pla*_, and WT, respectively. Finally, 20 037 genes whose average number of raw reads was >10 in all samples were used for further analysis.

### Statistical analysis

The statistical significance of differences between datasets with a minimum of three independent biological replicates was determined using one‐way analysis of variance (ANOVA) and Tukey's post hoc analysis (GraphPad Prism 9) or an unpaired Student's *t*‐test (Microsoft Excel 2016). *P*‐values ≤0.05, ≤ 0.01, ≤0.001, and ≤0.0001 are indicated by *, **, ***, and ****, respectively, and are considered to be significant.

Statistical analysis of RNA‐seq data were performed using R software version 4.1.2 (R Core Team, 2021). Gene Ontology (GO) enrichment analysis and Gene set enrichment analysis (GSEA) for GO and Kyoto Encyclopedia of Genes and Genomes (KEGG) (Kanehisa & Goto, n.d.) pathways were conducted as described previously (Reimand et al., 2019) with a Q‐value cut‐off of 0.05 using the R package GO. db version 3.13.0 (Carlson, 2021), KEGGREST version 1.32.0 (Dan Tenenbaum, 2021), the R package clusterProfiler version 4.0.5 (Wu et al., 2021), Cytoscape version 3.9.0 (Shannon et al., 2003), and EnrichmentMap version 3.3 (Merico et al., 2010). Heatmaps were drawn using the R package ComplexHeatmap version 2.8.0 (Gu et al., 2016).

## Author Contributions

KN conceived the original research plan; SRM‐Y generated the nucleus‐ and plastid‐encoded MaSp1 lines, performed screening of the mutants used in the study, designed and performed the experiments with support from CPF, CT, and MO^1^; YH performed the RNA‐seq data analysis; MO^4^ performed the ABA assay, water loss assay, and leaf temperature analysis; TS performed the LC–MS analysis; KN and MO^1^ supervised the study; SRM‐Y analyzed the data and wrote the article with contributions from all the authors. All authors read and approved the final manuscript.

## Conflicts of Interest

The authors declare that the research was conducted in the absence of any commercial or financial relationships that could be construed as a potential conflict of interest.

## Supporting information


**Figure S1.** Cloning method used for the generation of working constructs.
**Figure S2.** Confirmation of MaSp1 in *MaSp1*‐tobacco and cleavage of the N‐terminal His‐tag in *MaSp1*_*pla*_.
**Figure S3.** Chloroplast localization of MaSp1 in *MaSp1*_*pla*_ #2–2 and MaSp1 yield in *MaSp1*‐tobacco.
**Figure S4.** Growth analysis of *MaSp1*‐tobacco.
**Figure S5.** Leaf moisture contents of leaf cut‐outs used for the tensile test.
**Figure S6.** Workflow of sample preparation for tensile testing of tobacco leaves.
**Figure S7.** Original constructs used in this study.
**Figure S8.** Plant growth characteristics of WT and *MaSp1*‐tobacco used for tensile testing.
**Figure S9.** Dry weights of *MaSp1*‐tobacco after recovery from drought stress (ψ_w_ − 1.42 MPa) and under unstressed conditions at the same growth stage.
**Figure S10.** GO and KEGG analyses of differentially expressed genes in *MaSp1*‐tobacco.
**Figure S11.** Transcriptional regulation of ABA‐responsive genes in unstressed *MaSp1*‐tobacco.
**Figure S12.** Prediction analyses of protein, DNA and RNA binding ability, and phase separation ability of MaSp1.
**Table S1.** List of genes shown in Figure 4a
**Table S2.** List of genes shown in Figure 6a
**Table S3.** List of primer sequences used in this study.
**Dataset S1.** List of proteins and peptides identified by LC–MS/MS.

## Data Availability

All data supporting the findings of this study are available within this article. The mass spectrometry proteomics data have been deposited in the ProteomeXchange Consortium (http://proteomecentral.proteomexchange.org) via the PRIDE partner repository (Vizcaíno et al., 2012) with the dataset identifiers PXD040180 and 10.6019/PXD040180. The RNA‐seq datasets generated and/or used in this study are available in the Sequence Read Archive (SRA) under accession number PRJNA937325.
