# Supplementary material for: Expression of spider silk protein in tobacco improves drought tolerance with minimal effects on its mechanotype
Source: Plant J. 2025 Jan 27;121(2):e17213. doi: 10.1111/tpj.17213 (PMC11771620; doi:10.1111/tpj.17213)
Supplement: Supplementary file 1 — Figure S1. Cloning method used for the generation of working constructs. Figure S2. Confirmation of MaSp1 in MaSp1‐tobacco and cleavage of the N‐terminal His‐tag in MaSp1 pla . Figure S3. Chloroplast localization of MaSp1 in MaSp1 pla #2–2 and MaSp1 yield in MaSp1‐tobacco. Figure S4. Growth analysis of MaSp1‐tobacco. Figure S5. Leaf moisture contents of leaf cut‐outs used for the tensile test. Figure S6. Workflow of sample preparation for tensile testing of tobacco leaves. Figure S7. Original constructs used in this study. Figure S8. Plant growth characteristics of WT and MaSp1‐tobacco used for tensile testing. Figure S9. Dry weights of MaSp1‐tobacco after recovery from drought stress (ψw − 1.42 MPa) and under unstressed conditions at the same growth stage. Figure S10. GO and KEGG analyses of differentially expressed genes in MaSp1‐tobacco. Figure S11. Transcriptional regulation of ABA‐responsive genes in unstressed MaSp1‐tobacco. Figure S12. Prediction analyses of protein, DNA and RNA binding ability, and phase separation ability of MaSp1. Table S1. List of genes shown in Figure 4a Table S2. List of genes shown in Figure 6a Table S3. List of primer sequences used in this study. Dataset S1. List of proteins and peptides identified by LC–MS/MS. [file TPJ-121-0-s001.zip › Supplementary information.pdf]

Supporting Information for

Expression of spider silk protein in tobacco improves drought tolerance with minimal effects on its mechanotype

Shamitha Rao Morey-Yagi[1,2], Yoichi Hashida[3], Masanori Okamoto[4], Masaki Odahara[1], Takehiro Suzuki[5], Chonprakun Thagun[2], Choon Pin Foong[2], Keiji Numata[1,2*]

*Corresponding author: Keiji Numata

Email: numata.keiji.3n@kyoto-u.ac.jp

This file includes the following supplemental information[#]:

Figures S1 to S12

Supplementary references

[#]Dataset 1, Tables S1, S2 and S3 are provided as excel files

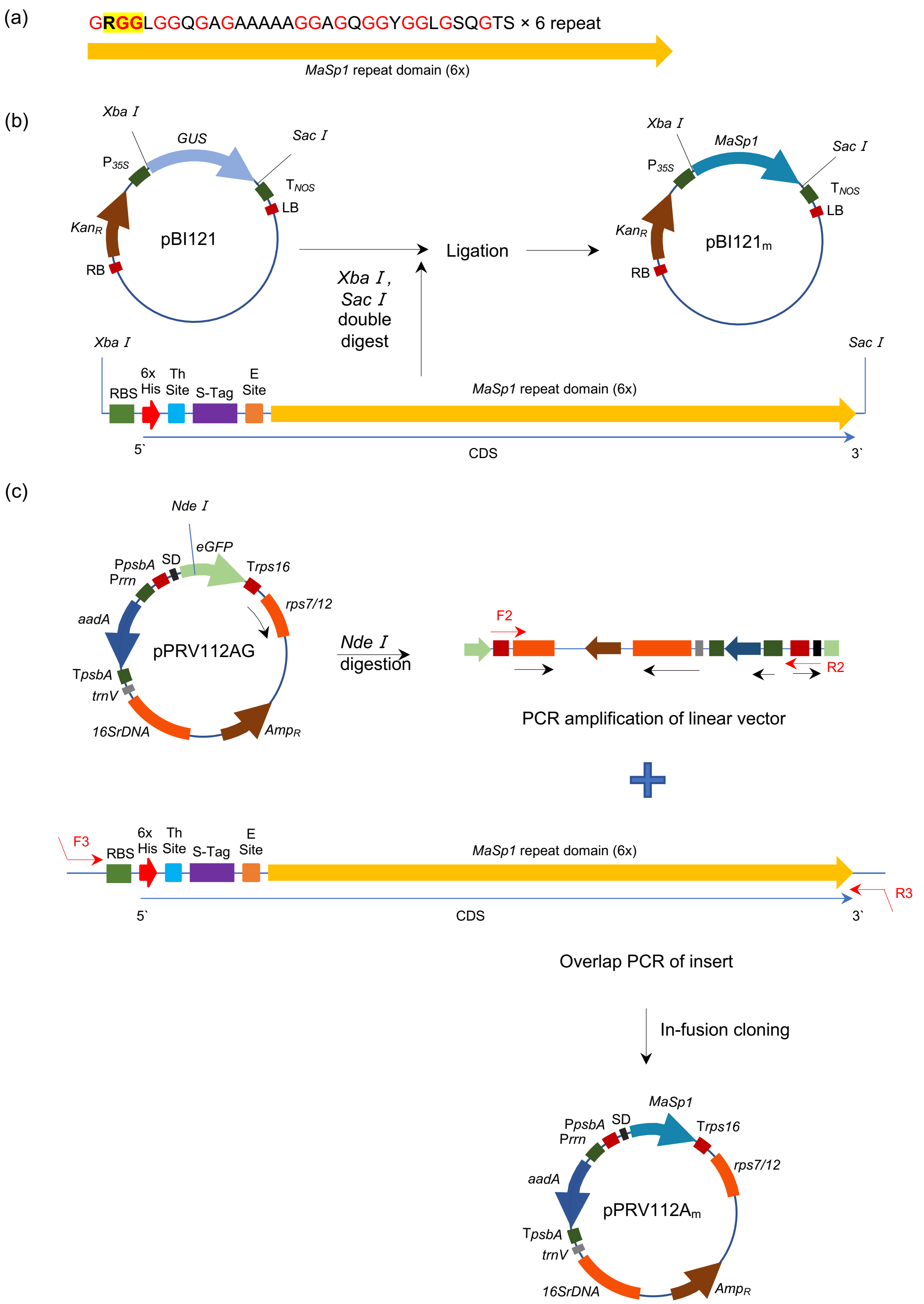

Figure S1. Cloning method used for the generation of working constructs.

(*a*) Amino acid sequence of the single repeat unit of the *MaSp1* hexamer, with the RGG sequence highlighted in yellow and shown in bold. Glycine residues are indicated in red. *MaSp1* was introduced into the (*b*) pBI121 and used for nuclear transformation, and (*c*) pPRV112AG and used for plastid transformation of *N. tabacum*. Red arrows indicate the primers; CDS, coding sequence.

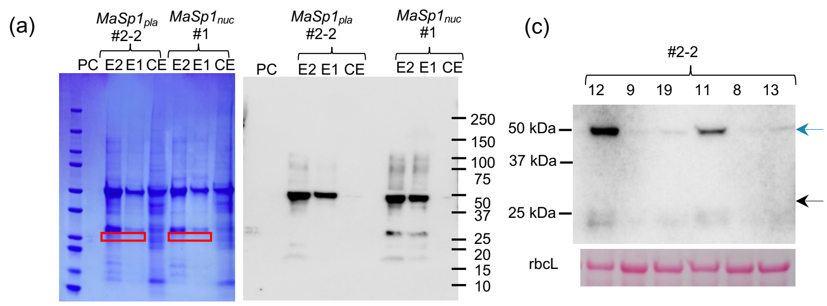

(b)

```
MHH HHH HSS GLV PRG SGM KET AAA KFE RQH MDS PDL GTD DDD KAM AAS

GRG GLG GQG AGA AAA AGG AGQ GGY GGL GSQ GTS GRG GLG GQG AGA AAA

AGG AGQ GGY GGL GSQ GTS GRG GLG GQG AGA AAA AGG AGQ GGY GGL GSQ

GTS GRG GLG GQG AGA AAA AGG AGQ GGY GGL GSQ GTS GRG GLG GQG AGA

AAA AGG AGQ GGY GGL GSQ GTS GRG GLG GQG AGA AAA AGG AGQ GGY GGL

GSQ GTS

Coverage: 100%
```

(d)

```
MHH HHH HSS GLV PRG SGM KET AAA KFE RQH MDS PDL GTD DDD KAM AAS

GRG GLG GQG AGA AAA AGG AGQ GGY GGL GSQ GTS GRG GLG GQG AGA AAA

AGG AGQ GGY GGL GSQ GTS GRG GLG GQG AGA AAA AGG AGQ GGY GGL GSQ

GTS GRG GLG GQG AGA AAA AGG AGQ GGY GGL GSQ GTS GRG GLG GQG AGA

AAA AGG AGQ GGY GGL GSQ GTS GRG GLG GQG AGA AAA AGG AGQ GGY GGL

GSQ GTS

Coverage: 67.07%
```

(e)

chlorop v1.1 prediction results

Name	Length	Score	cTP	CS-score	cTP length
MaSp1	246	0.444	–	5.938	45

(f)

```
5′ AGG GGA ATT GTG AGC GGA TAA CAA TTC CCC TCT AGA AAT AAT TTT GTT

TAA CTT TAA GAA GGA GAT ATA CAT ATG CAC CAT CAT CAT CAT CAT TCT
                               Met His His His His His His Ser

TCT GGT CTG GTG CCA CGC GGT TCT GGT ATG AAA GAA ACC GCT GCT GCT
Ser Gly Leu Val Pro Arg Gly Ser Gly Met Lys Glu Thr Ala Ala Ala

AAA TTC GAA CGC CAG CAC ATG GAC AGC CCA GAT CTG GGT ACC GAC GAC
Lys Phe Glu Arg Gln His Met Asp Ser Pro Asp Leu Gly Thr Asp Asp

GAC GAC AAG GCC ATG GCT GCT AGC GGT 3′
Asp Asp Lys Ala Met Ala Ala Ser Gly
```

Figure S2. Confirmation of MaSp1 in *MaSp1*-tobacco and cleavage of the N-terminal His-tag in *MaSp1*$_{pla}$.

(*a*) SDS-PAGE and immunoblotting of His-trap purified proteins from *MaSp1*$_{nuc}$#1 and *MaSp1*$_{pla}$#2-2 plants. Ten micrograms each of crude extract (CE), His-Trap purified fractions (E1 and E2), and the positive control (PC) were loaded into each lane. Red boxes indicate the bands excised and analyzed by LC-MS/MS. *(b)* shows the sequence coverage of the identified peptide in *MaSp1*$_{nuc}$#1. Protein expression in the T1 progeny of *MaSp1*$_{pla}$#2-2 was confirmed by (*c*) immunoblotting of 15 µg of TSP per well using a monoclonal anti-6xHis antibody. Dimerized MaSp1 is indicated by a blue arrow. *(d)* shows the sequence coverage of the identified peptide in *MaSp1*$_{pla}$#2-2. The confirmed sequence is shown in grey. (*e*) Peptide cleavage at position 45 predicted using chloroP v1.1 software, as (*f*) indicated in the MaSp1 sequence introduced in *MaSp1*$_{pla}$. Red arrow indicates the cleavage site.

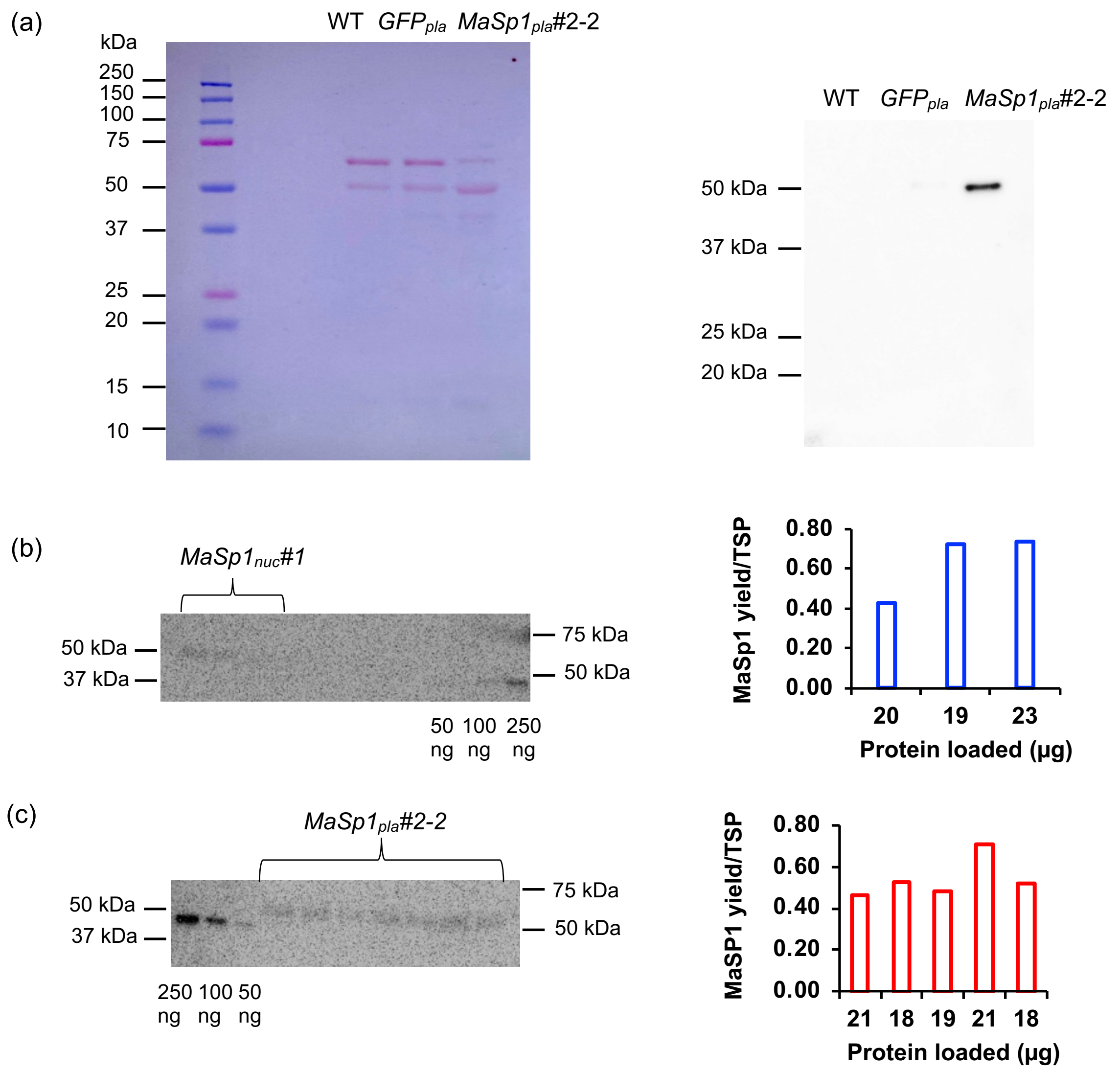

Figure S3. Confirmation of the chloroplast localization of MaSp1 in *MaSp1*$_{pla}$#2-2 and evaluation of MaSp1 yield in *MaSp1*-tobacco.

(*a*) The chloroplast localization of MaSp1 was confirmed by immunoblotting of five micrograms total soluble chloroplast protein per lane. Ponceau staining (left panel) to confirm equal lane loading and immunoblotting (right panel) of the soluble protein fraction from isolated WT, *GFP*$_{pla}$ and *MaSp1*$_{pla}$#2-2 chloroplasts using an anti-His tag antibody was performed. (*b*),(*c*) MaSp1 protein yield in (*b*) *MaSp1*$_{nuc}$#1 and (*c*) *MaSp1*$_{pla}$#2-2 was evaluated by immunoblotting (using an anti-His tag antibody) of 18 to 23 µg of total soluble protein (TSP) per lane together with 50 ng, 100 ng and 250 ng of known-His-tagged protein as a control (left panel), and estimation of protein content based on band intensity using ImageJ 1.52q (right panel).

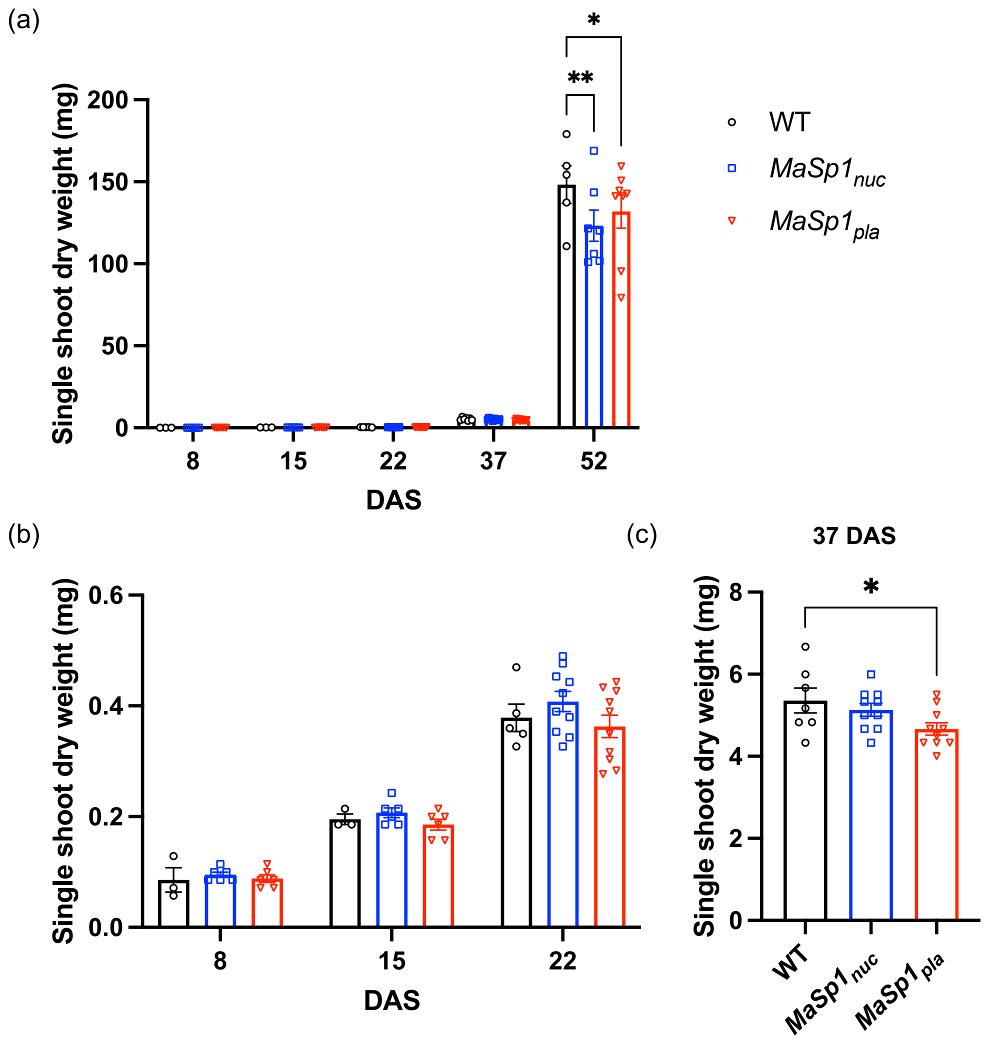

Figure S4. Growth analysis of *MaSp1*-tobacco from 8 DAS to 52 DAS.

(*a*) Single shoot dry weights for WT, $MaSp1_{nuc}$(#1 and #3), and $MaSp1_{pla}$(#2-2-8 and #2-2-9) measured at 8 DAS (n=3 to 6 sets, 7 plants each set), 15 DAS (n=3 to 6 sets, 7 plants each set), 22 DAS (n=5 to 10 sets, 3 plants each set), 37 DAS (n=7 to 10 sets, 3 plants each set) and 52 DAS (n=5 plants). Statistically significant differences between the genotypes over the growth period were evaluated by a two-way ANOVA (Tukey's test). Single shoot dry weights at (*b*) 8 DAS, 15 DAS, 22 DAS, and (*c*) 37 DAS are shown; statistically significant differences with the WT at each growth stage were evaluated by a one-way ANOVA (Dunnett's test). Data represent means±SEM and asterisks indicate statistically significant differences at * $p<0.05$ and ** $p<0.01$.

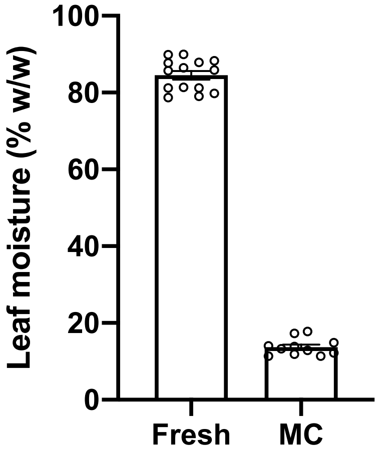

Figure S5. Leaf moisture contents of leaf cut-outs (fresh and moisture conditioned; MC) used for the tensile test.

Data represent means±SEM of n=11 or 14 samples from 4 independent plants.

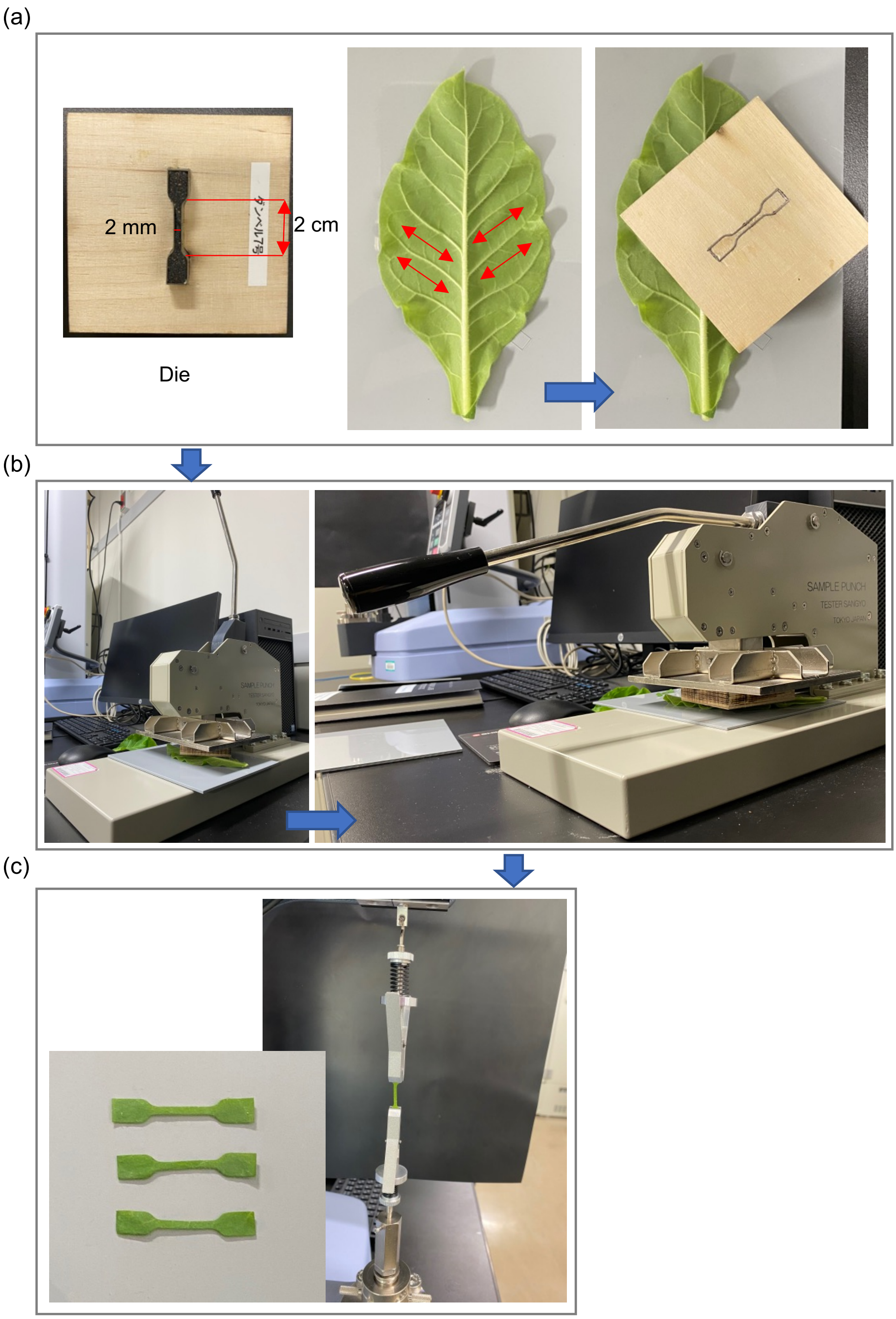

Figure S6. Workflow of sample preparation for tensile testing of tobacco leaves.

(*a*) Dimensions and placement of the die on the abaxial leaf surface to obtain leaf cut-outs, (*b*) sample punch set up for punching out leaf samples, and (*c*) leaf cut-outs used for the tensile test.

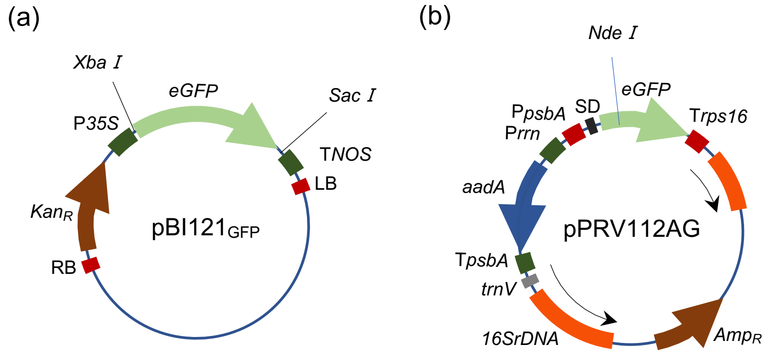

Figure S7. Original constructs used in this study.

Constructs used to generate the (*a*) nucleus-encoded and (*b*) plastid-encoded GFP lines.

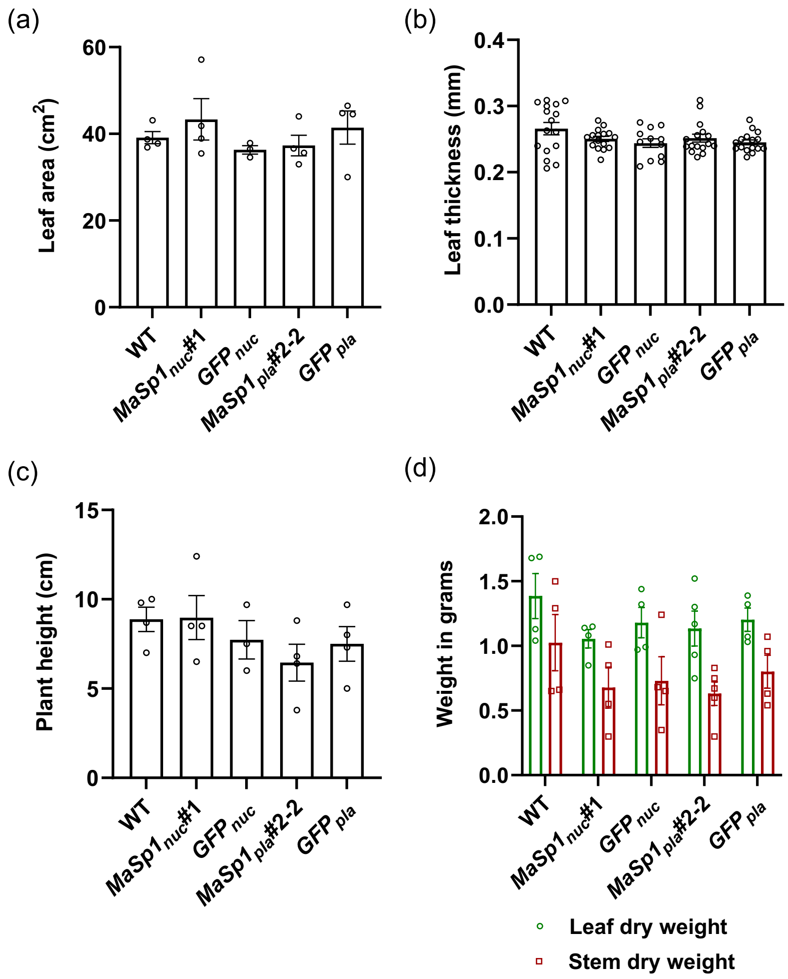

Figure S8. Plant growth characteristics of WT, nucleus-encoded lines (*MaSp1*$_{nuc}$ and *GFP*$_{nuc}$), and plastid-encoded lines (*MaSp1*$_{pla}$ and *GFP*$_{pla}$) used for tensile testing.

(*a*) Leaf area, (*b*) leaf thickness, (*c*) plant height, and (*d*) dry weights of representative lines from plants used for tensile testing. Data represent means±SEM of n=3 or 4 plants for (*a*, *c*, *d*), and n= 12 or 16 for (*b*). Significant differences between the genotypes were not observed as determined by a one-way ANOVA (Tukey's test).

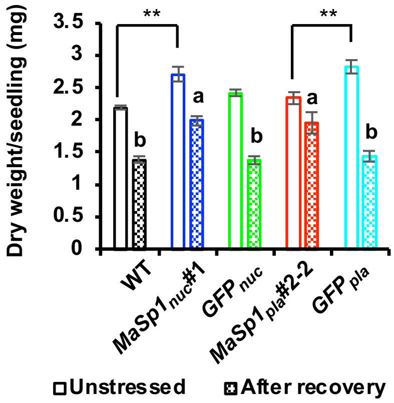

Figure S9. Dry weights of *MaSp1*-tobacco after recovery from drought stress (Ψ_w -1.42 MPa) and under unstressed conditions at the same growth stage.

Single seedling dry weight of WT, *MaSp1*-expressing plants (#1_{nuc} and #2-2_{pla}), and their corresponding *GFP* controls (*GFP_{nuc}* and *GFP_{pla}*) at 2 DAR from drought stress (after recovery) and at 22 DAS in MS medium (unstressed). Data represent means±SEM of n=12 to 40 plants. Asterisks and different letters indicate statistically significant differences between the genotypes (** $p<0.01$) for 'unstressed' and 'after recovery' conditions respectively, and were evaluated by a two-way ANOVA (Tukey's test).

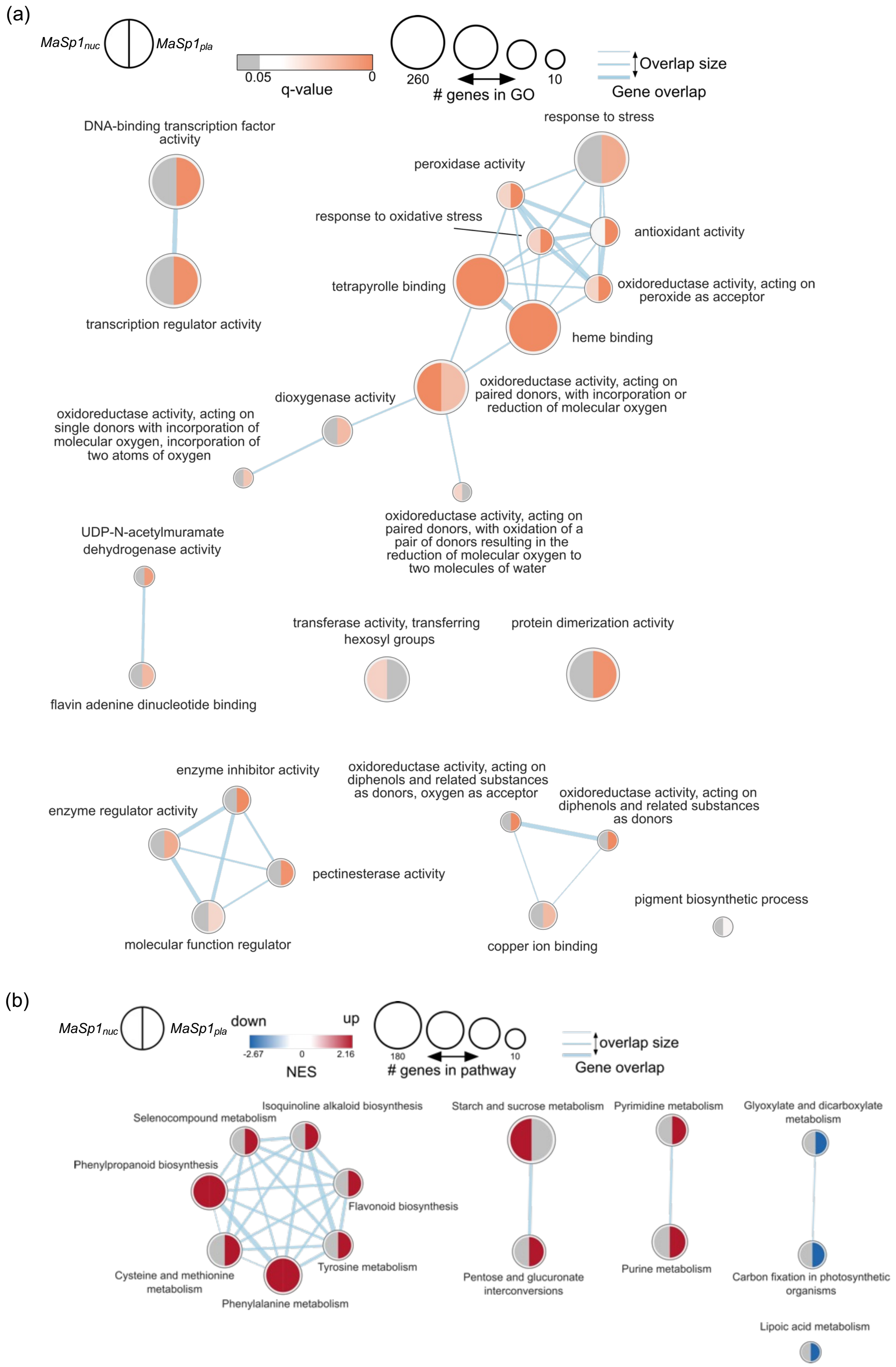

Figure S10. GO and KEGG analyses of differentially expressed genes in *MaSp1*$_{nuc}$ and *MaSp1*$_{pla}$ plants.

(*a*) Network analysis of fold changes of upregulated GO terms and (*b*) GSEA of KEGG terms, with more than two-fold differences compared to the WT. RNA-seq data were obtained for pooled RNA from n=8 plants for each genotype.

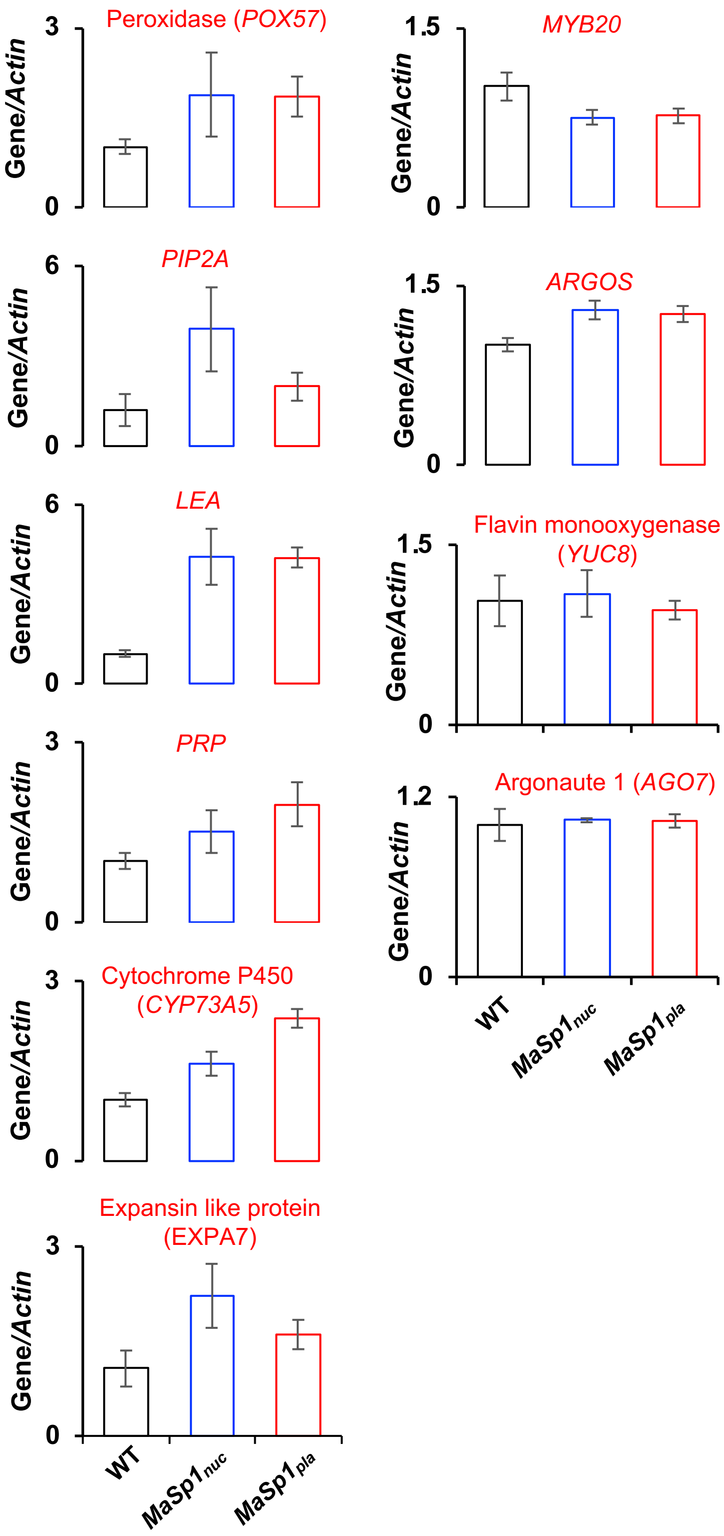

Figure S11. Transcriptional regulation of ABA-responsive genes in unstressed *MaSp1*-tobacco shown in Figure 6b.

RT-qPCR confirmation of few key players in drought tolerance (shown in red in Figure 6a), expressed as relative abundance to *Actin* in shoots from n=15 plants, where the gene/*Actin* ratio for WT was set to 1. Lipoxygenase (*LOX5*) transcripts were not detected in WT, hence the data is not shown. Data represent means±SEM.

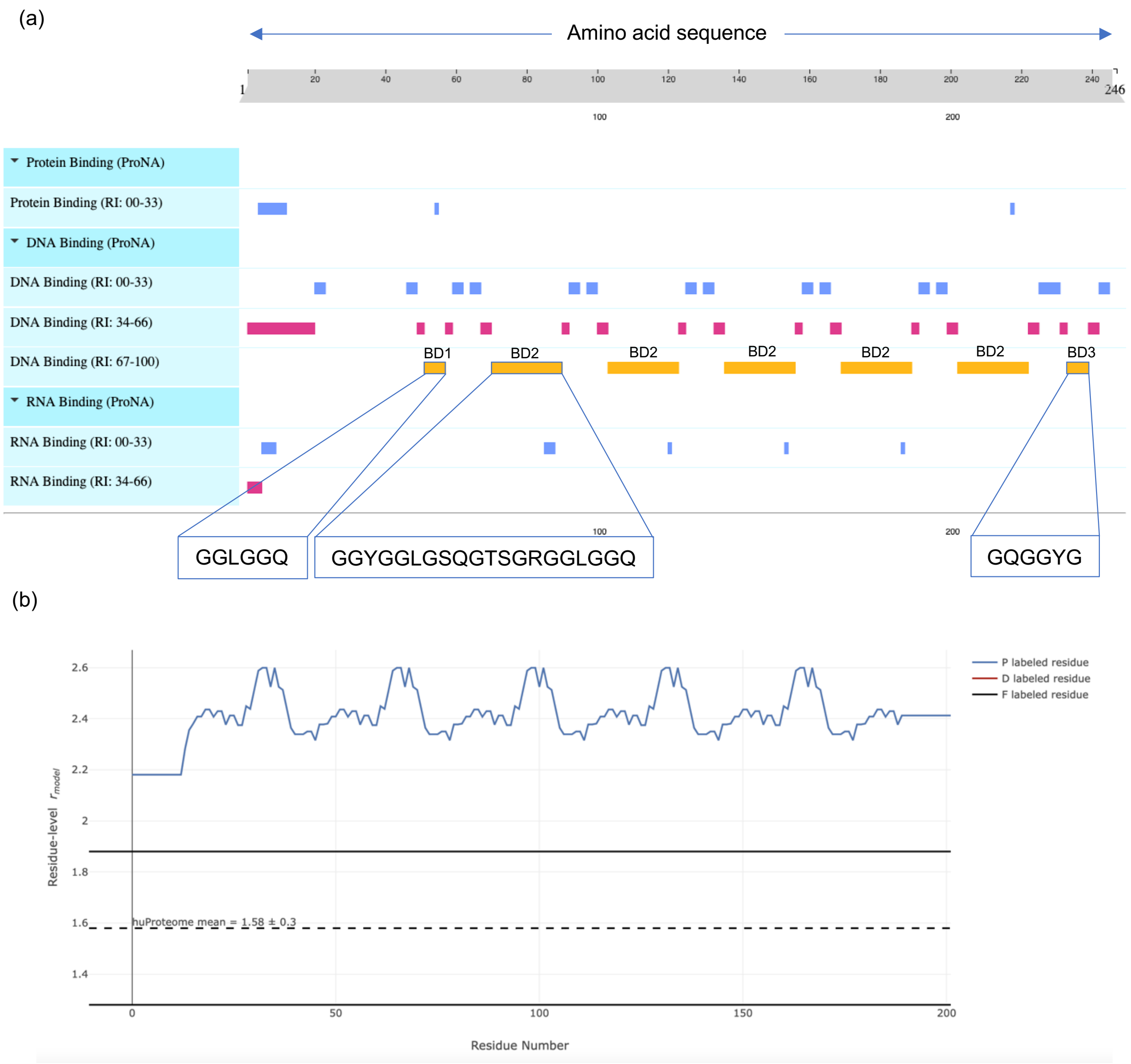

Figure S12. Prediction analyses of protein, DNA and RNA binding ability, and phase separation ability of MaSp1.

Amino acid sequence analysis using (*a*) ProNA2020 (Qui et al., 2020) to examine the protein, DNA, and RNA binding ability of MaSp1 and (*b*) ParSe (Paiz et al., 2021), an algorithm to predict the tendency of MaSp1 to undergo phase separation. In *(a)*, blue indicates a low-reliability score, pink indicates a medium-reliability score, and yellow indicates a high-reliability score for the prediction. RI indicates the reliability index or score. Blue open boxes show the amino acid sequences of the three predicted DNA binding domains (BDs). In *(b)*, P regions are intrinsically disordered and prone to undergo LLPS, D regions are intrinsically disordered but do not undergo LLPS, and F regions may or may not be intrinsically disordered but can fold into a stable conformation.

Supplementary references (For Table S2 that are not included in the manuscript)

Abe, H. *et al.* (2003) 'Arabidopsis AtMYC2 (bHLH) and AtMYB2 (MYB) Function as Transcriptional Activators in Abscisic Acid Signaling', *The Plant Cell*, 15(1), pp. 63–78. Available at: https://doi.org/10.1105/tpc.006130.

Abe, H. et al. (1997) 'Role of arabidopsis MYC and MYB homologs in drought- and abscisic acid-regulated gene expression', The Plant Cell, 9(10), 1859-68. Available at: https://doi.org/10.1105/tpc.9.10.1859.

Ahmed, I.M. *et al.* (2020) 'The Barley S-Adenosylmethionine Synthetase 3 Gene HvSAMS3 Positively Regulates the Tolerance to Combined Drought and Salinity Stress in Tibetan Wild Barley', *Cells*, 9(6), p. 1530. Available at: https://doi.org/10.3390/cells9061530.

Alazem, M. *et al.* (2017) 'Abscisic Acid Induces Resistance against *Bamboo Mosaic Virus* through *Argonaute 2* and *3*', *Plant Physiology*, 174(1), pp. 339–355. Available at: https://doi.org/10.1104/pp.16.00015.

Alexandersson, E. *et al.* (2005) 'Whole Gene Family Expression and Drought Stress Regulation of Aquaporins', *Plant Molecular Biology*, 59(3), pp. 469–484. Available at: https://doi.org/10.1007/s11103-005-0352-1.

Almira Casellas, M.J. *et al.* (2023) 'A genome-wide association study identifies novel players in Na and Fe homeostasis in *Arabidopsis thaliana* under alkaline-salinity stress', *The Plant Journal*, 113(2), pp. 225–245. Available at: https://doi.org/10.1111/tpj.16042.

Alzwiy, I.A. and Morris, P.C. (2007) 'A mutation in the Arabidopsis MAP kinase kinase 9 gene results in enhanced seedling stress tolerance', *Plant Science*, 173(3), pp. 302–308. Available at: https://doi.org/10.1016/j.plantsci.2007.06.007.

Ascencio-Ibáñez, J.T. *et al.* (2008) 'Global Analysis of Arabidopsis Gene Expression Uncovers a Complex Array of Changes Impacting Pathogen Response and Cell Cycle during Geminivirus Infection', *Plant Physiology*, 148(1), pp. 436–454. Available at: https://doi.org/10.1104/pp.108.121038.

Baek, D. *et al.* (2020) 'Histone Deacetylase HDA9 With ABI4 Contributes to Abscisic Acid Homeostasis in Drought Stress Response', *Frontiers in Plant Science*, 11, p. 143. Available at: https://doi.org/10.3389/fpls.2020.00143.

Bai, J. *et al.* (2021) 'Rice aquaporin OsPIP2;2 is a water-transporting facilitator in relevance to drought-tolerant responses', *Plant Direct*, 5(8), p. e338. Available at: https://doi.org/10.1002/pld3.338.

Balassa, G. *et al.* (2022) 'Expression Pattern of RNA Interference Genes During Drought Stress and MDMV Infection in Maize', *Journal of Plant Growth Regulation*, 41(5), pp. 2048–2058. Available at: https://doi.org/10.1007/s00344-022-10651-z.

Barquero, M. *et al.* (2022) 'Mechanisms involved in drought stress tolerance triggered by rhizobia strains in wheat', *Frontiers in Plant Science*, 13, p. 1036973. Available at: https://doi.org/10.3389/fpls.2022.1036973.

Ben-Hayyim, G. *et al.* (2001) 'Preferential induction of a 9-lipoxygenase by salt in salt-tolerant cells of Citrus sinensis L. Osbeck', *Planta*, 212(3), pp. 367–375. Available at: https://doi.org/10.1007/s004250000397.

Cai, T. *et al.* (2023) 'In-silico identification and characterization of O-methyltransferase gene family in peanut (Arachis hypogaea L.) reveals their putative roles in development and stress tolerance', *Frontiers in Plant Science*, 14, p. 1145624. Available at: https://doi.org/10.3389/fpls.2023.1145624.

Castañera, P., Steffens, J.C. and Tingey, W.M. (1996) 'Biological performance of Colorado potato beetle larvae on potato genotypes with differing levels of polyphenol oxidase', *Journal of Chemical*

Ecology, 22(1), pp. 91–101. Available at: https://doi.org/10.1007/BF02040202.

Chang, J. *et al.* (2021) ‘The role of watermelon caffeic acid O-methyltransferase (ClCOMT1) in melatonin biosynthesis and abiotic stress tolerance’, *Horticulture Research*, 8(1), p. 210. Available at: https://doi.org/10.1038/s41438-021-00645-5.

Chen, I. *et al.* (2003) ‘The transcriptional response of *Arabidopsis* to genotoxic stress – a high-density colony array study (HDCA)’, *The Plant Journal*, 35(6), pp. 771–786. Available at: https://doi.org/10.1046/j.1365-313X.2003.01847.x.

Chen, Q. *et al.* (2023) ‘An R2R3-MYB FtMYB11 from Tartary buckwheat has contrasting effects on abiotic tolerance in Arabidopsis’, *Journal of Plant Physiology*, 280, p. 153842. Available at: https://doi.org/10.1016/j.jplph.2022.153842.

Chen, S. *et al.* (2022) ‘Quantitative proteomics analysis of tomato root cell wall proteins in response to salt stress’, *Frontiers in Plant Science*, 13, p. 1023388. Available at: https://doi.org/10.3389/fpls.2022.1023388.

Chen, Y. *et al.* (2016) ‘Overexpression of the Wheat Expansin Gene TaEXPA2 Improved Seed Production and Drought Tolerance in Transgenic Tobacco Plants’, *PLOS ONE*. Edited by Z.M. Yang, 11(4), p. e0153494. Available at: https://doi.org/10.1371/journal.pone.0153494.

Cherepneva, G.N. *et al.* (2003) ‘Expression of the ribosomal proteins S14, S16, L13a and L30 is regulated by cytokinin and abscisic acid’, *Plant Science*, 165(5), pp. 925–932. Available at: https://doi.org/10.1016/S0168-9452(03)00204-8.

Chowdhury, E. *et al.* (2012) ‘Transcriptional analysis of hydroxycinnamoyl transferase (HCT) in various tissues of Hibiscus cannabinus in response to abiotic stress conditions’, 5(3), pp. 305–313.

Chun, H.J. *et al.* (2021) ‘Arabidopsis CCoAOMT1 Plays a Role in Drought Stress Response via ROS- and ABA-Dependent Manners’, *Plants*, 10(5), p. 831. Available at: https://doi.org/10.3390/plants10050831.

Comelli, R.N. and Gonzalez, D.H. (2009) ‘Identification of regulatory elements involved in expression and induction by sucrose and UV-B light of the *Arabidopsis thaliana COX5b-2* gene, encoding an isoform of cytochrome *c* oxidase subunit 5b’, *Physiologia Plantarum*, 137(3), pp. 213–224. Available at: https://doi.org/10.1111/j.1399-3054.2009.01285.x.

Cui, M.H. *et al.* (2013) ‘An Arabidopsis R2R3-MYB transcription factor, AtMYB20, negatively regulates type 2C serine/threonine protein phosphatases to enhance salt tolerance’, *FEBS Letters*, 587(12), pp. 1773–1778. Available at: https://doi.org/10.1016/j.febslet.2013.04.028.

Da Costa-Nunes, J.A. *et al.* (2006) ‘Characterization of the three Arabidopsis thaliana RAD21 cohesins reveals differential responses to ionizing radiation’, *Journal of Experimental Botany*, 57(4), pp. 971–983. Available at: https://doi.org/10.1093/jxb/erj083.

Dubois, M. *et al.* (2013) ‘ETHYLENE RESPONSE FACTOR6 Acts as a Central Regulator of Leaf Growth under Water-Limiting Conditions in Arabidopsis’, *Plant Physiology*, 162(1), pp. 319–332. Available at: https://doi.org/10.1104/pp.113.216341.

Eljebbawi, A. *et al.* (2022) ‘Class III Peroxidases in Response to Multiple Abiotic Stresses in Arabidopsis thaliana Pyrenean Populations’, *International Journal of Molecular Sciences*, 23(7), p. 3960. Available at: https://doi.org/10.3390/ijms23073960.

Fonseca, S. and Rubio, V. (2019) ‘Arabidopsis CRL4 Complexes: Surveying Chromatin States and Gene Expression’, *Frontiers in Plant Science*, 10, p. 1095. Available at: https://doi.org/10.3389/fpls.2019.01095.

Frost, J.M. *et al.* (2023) ‘H2A.X promotes endosperm-specific DNA methylation in Arabidopsis

thaliana'. Available at: https://doi.org/10.21203/rs.3.rs-2974671/v1.

Fujita, K. *et al.* (2022) 'MLP-PG1, a major latex-like protein identified in Cucurbita pepo, confers resistance through the induction of pathogenesis-related genes', *Planta*, 255(1), p. 10. Available at: https://doi.org/10.1007/s00425-021-03795-x.

Ghifari, A.S. *et al.* (2020) 'A mitochondrial prolyl aminopeptidase PAP2 releases N-terminal proline and regulates proline homeostasis during stress response', *The Plant Journal*, 104(5), pp. 1182–1194. Available at: https://doi.org/10.1111/tpj.14987.

Goda, H. *et al.* (2004) 'Comprehensive Comparison of Auxin-Regulated and Brassinosteroid-Regulated Genes in Arabidopsis', *Plant Physiology*, 134(4), pp. 1555–1573. Available at: https://doi.org/10.1104/pp.103.034736.

Gong, B. *et al.* (2014) 'Overexpression of S-adenosyl- L -methionine synthetase increased tomato tolerance to alkali stress through polyamine metabolism', *Plant Biotechnology Journal*, 12(6), pp. 694–708. Available at: https://doi.org/10.1111/pbi.12173.

Gottardini, E. *et al.* (2016) 'Suppression Substractive Hybridization and NGS Reveal Differential Transcriptome Expression Profiles in Wayfaring Tree (Viburnum lantana L.) Treated with Ozone', *Frontiers in Plant Science*, 7. Available at: https://doi.org/10.3389/fpls.2016.00713.

Guo, Y. and Gan, S. (2006) 'AtNAP, a NAC family transcription factor, has an important role in leaf senescence', *The Plant Journal*, 46(4), pp. 601–612. Available at: https://doi.org/10.1111/j.1365-313X.2006.02723.x.

Gupta, P. *et al.* (2017) 'Abiotic Stresses Cause Differential Regulation of Alternative Splice Forms of GATA Transcription Factor in Rice', *Frontiers in Plant Science*, 8, p. 1944. Available at: https://doi.org/10.3389/fpls.2017.01944.

Han, Y. *et al.* (2015) 'Over-expression of TaEXPB23, a wheat expansin gene, improves oxidative stress tolerance in transgenic tobacco plants', *Journal of Plant Physiology*, 173, pp. 62–71. Available at: https://doi.org/10.1016/j.jplph.2014.09.007.

Henry, E. *et al.* (2015) 'Beyond Glycolysis: GAPDHs Are Multi-functional Enzymes Involved in Regulation of ROS, Autophagy, and Plant Immune Responses', *PLOS Genetics*. Edited by J.M. McDowell, 11(4), p. e1005199. Available at: https://doi.org/10.1371/journal.pgen.1005199.

Hou, Y. *et al.* (2023) 'A Cinnamate 4-HYDROXYLASE1 from Safflower Promotes Flavonoids Accumulation and Stimulates Antioxidant Defense System in Arabidopsis', *International Journal of Molecular Sciences*, 24(6), p. 5393. Available at: https://doi.org/10.3390/ijms24065393.

Huang, E. *et al.* (2024) 'Caffeic acid O-methyltransferase from Ligusticum chuanxiong alleviates drought stress, and improves lignin and melatonin biosynthesis', *Frontiers in Plant Science*, 15, p. 1458296. Available at: https://doi.org/10.3389/fpls.2024.1458296.

Huang, X. *et al.* (2010) 'The *Arabidopsis LSD1* gene plays an important role in the regulation of low temperature-dependent cell death', *New Phytologist*, 187(2), pp. 301–312. Available at: https://doi.org/10.1111/j.1469-8137.2010.03275.x.

Ismond, K.P. *et al.* (2003) 'Enhanced Low Oxygen Survival in Arabidopsis through Increased Metabolic Flux in the Fermentative Pathway', *Plant Physiology*, 132(3), pp. 1292–1302. Available at: https://doi.org/10.1104/pp.103.022244.

Iuchi, S. *et al.* (2001) 'Regulation of drought tolerance by gene manipulation of 9- *cis* -epoxycarotenoid dioxygenase, a key enzyme in abscisic acid biosynthesis in *Arabidopsis*', *The Plant Journal*, 27(4), pp. 325–333. Available at: https://doi.org/10.1046/j.1365-313x.2001.01096.x.

Jiang, Y. and Deyholos, M.K. (2009) 'Functional characterization of Arabidopsis NaCl-inducible

WRKY25 and WRKY33 transcription factors in abiotic stresses', *Plant Molecular Biology*, 69(1–2), pp. 91–105. Available at: https://doi.org/10.1007/s11103-008-9408-3.

Kavitha, R. and Umesha, S. (2008) 'Regulation of defense-related enzymes associated with bacterial spot resistance in Tomato', *Phytoparasitica*, 36(2), pp. 144–159. Available at: https://doi.org/10.1007/BF02981327.

Kim, K.-J. *et al.* (2006) 'Induction of a cytosolic pyruvate kinase 1 gene during the resistance response to Tobacco mosaic virus in Capsicum annuum', *Plant Cell Reports*, 25(4), pp. 359–364. Available at: https://doi.org/10.1007/s00299-005-0082-5.

Kim, K.-Y. (2004) 'Molecular cloning of low-temperature-inducible ribosomal proteins from soybean', *Journal of Experimental Botany*, 55(399), pp. 1153–1155. Available at: https://doi.org/10.1093/jxb/erh125.

Kim, Sun Hee *et al.* (2015) 'Expression of potato S-adenosyl-l-methionine synthase (SbSAMS) gene altered developmental characteristics and stress responses in transgenic Arabidopsis plants', *Plant Physiology and Biochemistry*, 87, pp. 84–91. Available at: https://doi.org/10.1016/j.plaphy.2014.12.020.

Kline, K.G., Barrett-Wilt, G.A. and Sussman, M.R. (2010) '*In planta* changes in protein phosphorylation induced by the plant hormone abscisic acid', *Proceedings of the National Academy of Sciences*, 107(36), pp. 15986–15991. Available at: https://doi.org/10.1073/pnas.1007879107.

Klinkenberg, J. *et al.* (2014) 'Two Fatty Acid Desaturases, STEAROYL-ACYL CARRIER PROTEIN Δ^{9} -DESATURASE6 and FATTY ACID DESATURASE3, Are Involved in Drought and Hypoxia Stress Signaling in Arabidopsis Crown Galls', *Plant Physiology*, 164(2), pp. 570–583. Available at: https://doi.org/10.1104/pp.113.230326.

Ko, J.-H. *et al.* (2011) 'Novel aspects of transcriptional regulation in the winter survival and maintenance mechanism of poplar', *Tree Physiology*, 31(2), pp. 208–225. Available at: https://doi.org/10.1093/treephys/tpq109.

Kruse, L.H. *et al.* (2023) 'Phylogenomic analyses across land plants reveals motifs and coexpression patterns useful for functional prediction in the BAHD acyltransferase family', *Frontiers in Plant Science*, 14, p. 1067613. Available at: https://doi.org/10.3389/fpls.2023.1067613.

Kushiro, T. *et al.* (2004) 'The Arabidopsis cytochrome P450 CYP707A encodes ABA 8′-hydroxylases: key enzymes in ABA catabolism', *The EMBO Journal*, 23(7), pp. 1647–1656. Available at: https://doi.org/10.1038/sj.emboj.7600121.

Lai, Z. *et al.* (2011) '*Arabidopsis* Sigma Factor Binding Proteins Are Activators of the WRKY33 Transcription Factor in Plant Defense', *The Plant Cell*, 23(10), pp. 3824–3841. Available at: https://doi.org/10.1105/tpc.111.090571.

Lee, Y., Choi, D. and Kende, H. (2001) 'Expansins: ever-expanding numbers and functions', *Current Opinion in Plant Biology*, 4(6), pp. 527–532. Available at: https://doi.org/10.1016/S1369-5266(00)00211-9.

Li, F. *et al.* (2011) 'Drought tolerance through over-expression of the expansin gene TaEXPB23 in transgenic tobacco', *Journal of Plant Physiology*, 168(9), pp. 960–966. Available at: https://doi.org/10.1016/j.jplph.2010.11.023.

Lim, J., Lim, C.W. and Lee, S.C. (2018) 'The Pepper Late Embryogenesis Abundant Protein, CaDIL1, Positively Regulates Drought Tolerance and ABA Signaling', *Frontiers in Plant Science*, 9, p. 1301. Available at: https://doi.org/10.3389/fpls.2018.01301.

Liu, B. *et al.* (2017) 'Both AtrbohD and AtrbohF are essential for mediating responses to oxygen deficiency in Arabidopsis', *Plant Cell Reports*, 36(6), pp. 947–957. Available at: https://doi.org/10.1007/s00299-017-2128-x.

Liu, J. *et al.* (2020) ‘Involvement of active MKK9-MAPK3/MAPK6 in increasing respiration in salt-treated Arabidopsis callus’, *Protoplasma*, 257(3), pp. 965–977. Available at: https://doi.org/10.1007/s00709-020-01483-3.

Liu, M. *et al.* (2023) ‘Regulation of drought tolerance in *Arabidopsis* involves the PLATZ4 -mediated transcriptional repression of plasma membrane aquaporin *PIP2;8*’, *The Plant Journal*, 115(2), pp. 434–451. Available at: https://doi.org/10.1111/tpj.16235.

Liu, S. *et al.* (2015) ‘Negative regulation of ABA signaling by WRKY33 is critical for Arabidopsis immunity towards Botrytis cinerea 2100’, *eLife*, 4, p. e07295. Available at: https://doi.org/10.7554/eLife.07295.

Liu, W. *et al.* (2024) ‘Characterization of the pyruvate kinase gene family in soybean and identification of a putative salt responsive gene GmPK21’, *BMC Genomics*, 25(1), p. 88. Available at: https://doi.org/10.1186/s12864-023-09929-7.

Liu, Y. and Imai, R. (2018) ‘Function of Plant DExD/H-Box RNA Helicases Associated with Ribosomal RNA Biogenesis’, *Frontiers in Plant Science*, 9, p. 125. Available at: https://doi.org/10.3389/fpls.2018.00125.

López, M.A. *et al.* (2011) ‘Antagonistic role of 9-lipoxygenase-derived oxylipins and ethylene in the control of oxidative stress, lipid peroxidation and plant defence’, *The Plant Journal*, 67(3), pp. 447–458. Available at: https://doi.org/10.1111/j.1365-313X.2011.04608.x.

Lorković, Z.J. *et al.* (2017) ‘Compartmentalization of DNA Damage Response between Heterochromatin and Euchromatin Is Mediated by Distinct H2A Histone Variants’, *Current Biology*, 27(8), pp. 1192–1199. Available at: https://doi.org/10.1016/j.cub.2017.03.002.

Luo, M. *et al.* (2012) ‘HD2C interacts with HDA6 and is involved in ABA and salt stress response in Arabidopsis’, *Journal of Experimental Botany*, 63(8), pp. 3297–3306. Available at: https://doi.org/10.1093/jxb/ers059.

Ma, C. *et al.* (2017) ‘Overexpression of S-Adenosyl-l-Methionine Synthetase 2 from Sugar Beet M14 Increased Arabidopsis Tolerance to Salt and Oxidative Stress’, *International Journal of Molecular Sciences*, 18(4), p. 847. Available at: https://doi.org/10.3390/ijms18040847.

Ma, S. and Bohnert, H.J. (2007) ‘Integration of Arabidopsis thaliana stress-related transcript profiles, promoter structures, and cell-specific expression’, *Genome Biology*, 8(4), p. R49. Available at: https://doi.org/10.1186/gb-2007-8-4-r49.

Mangano, S. *et al.* (2017) ‘Molecular link between auxin and ROS-mediated polar growth’, *Proceedings of the National Academy of Sciences*, 114(20), pp. 5289–5294. Available at: https://doi.org/10.1073/pnas.1701536114.

Manova, V. and Gruszka, D. (2015) ‘DNA damage and repair in plants – from models to crops’, *Frontiers in Plant Science*, 6, 885. Available at: https://doi.org/10.3389/fpls.2015.00885.

Martínez-Andújar, C. *et al.* (2021) ‘Overproduction of ABA in rootstocks alleviates salinity stress in tomato shoots’, *Plant, Cell & Environment*, 44(9), pp. 2966–2986. Available at: https://doi.org/10.1111/pce.14121.

Maruyama, K. *et al.* (2014) ‘Integrated Analysis of the Effects of Cold and Dehydration on Rice Metabolites, Phytohormones, and Gene Transcripts’, *Plant Physiology*, 164(4), pp. 1759–1771. Available at: https://doi.org/10.1104/pp.113.231720.

McLaughlin, J.E. *et al.* (2015) ‘A Lipid Transfer Protein Increases the Glutathione Content and Enhances Arabidopsis Resistance to a Trichothecene Mycotoxin’, *PLOS ONE*. Edited by E.J. Van Damme, 10(6), p. e0130204. Available at: https://doi.org/10.1371/journal.pone.0130204.

Merlot, S. *et al.* (2001) 'The ABI1 and ABI2 protein phosphatases 2C act in a negative feedback regulatory loop of the abscisic acid signalling pathway', *The Plant Journal*, 25(3), pp. 295–303. Available at: https://doi.org/10.1046/j.1365-313x.2001.00965.x.

Mizutani, M., Ohta, D. and Sato, R. (1997) 'Isolation of a cDNA and a Genomic Clone Encoding Cinnamate 4-Hydroxylase from Arabidopsis and Its Expression Manner in Planta', 113, pp. 755–763.

Mohammadi, M. and Kazemi, H. (2002) 'Changes in peroxidase and polyphenol oxidase activities in susceptible and resistant wheat heads inoculated with Fusarium graminearum and induced resistance', *Plant Science*, 162(4), pp. 491–498. Available at: https://doi.org/10.1016/S0168-9452(01)00538-6.

Molinari, M.D.C. *et al.* (2020) 'Overexpression of AtNCED3 gene improved drought tolerance in soybean in greenhouse and field conditions', *Genetics and Molecular Biology*, 43(3), p. e20190292. Available at: https://doi.org/10.1590/1678-4685-gmb-2019-0292.

Moradi, K. and Khalili, F. (2018) 'Assessment of pattern expression of miR172 and miR169 in response to drought stress in Echinacea purpurea L.', *Biocatalysis and Agricultural Biotechnology*, 16, pp. 507–512. Available at: https://doi.org/10.1016/j.bcab.2018.08.022.

Mukhopadhyay, P. and Tyagi, A.K. (2015) 'OsTCP19 influences developmental and abiotic stress signaling by modulatingABI4-mediated pathways', *Scientific Reports*, 5(1), p. 9998. Available at: https://doi.org/10.1038/srep09998.

Muñoz-Bertomeu, J. *et al.* (2009) 'Plastidial Glyceraldehyde-3-Phosphate Dehydrogenase Deficiency Leads to Altered Root Development and Affects the Sugar and Amino Acid Balance in Arabidopsis', *Plant Physiology*, 151(2), pp. 541–558. Available at: https://doi.org/10.1104/pp.109.143701.

Nagy, S.K. *et al.* (2015) 'Activation of AtMPK9 through autophosphorylation that makes it independent of the canonical MAPK cascades', *Biochemical Journal*, 467(1), pp. 167–175. Available at: https://doi.org/10.1042/BJ20141176.

Negi, J. *et al.* (2013) 'A Dof Transcription Factor, SCAP1, Is Essential for the Development of Functional Stomata in Arabidopsis', *Current Biology*, 23(6), pp. 479–484. Available at: https://doi.org/10.1016/j.cub.2013.02.001.

Niggeweg, R., Michael, A.J. and Martin, C. (2004) 'Engineering plants with increased levels of the antioxidant chlorogenic acid', *Nature Biotechnology*, 22(6), pp. 746–754. Available at: https://doi.org/10.1038/nbt966.

Nishiuchi, T. *et al.* (2002) 'Wounding activates immediate early transcription of genes for ERFs in tobacco plants', *Plant Molecular Biology,* 49, pp. 473–482. Available at: https://doi.org/10.1023/A:1015553232309

Nishizawa, A. *et al.* (2006) 'Arabidopsis heat shock transcription factor A2 as a key regulator in response to several types of environmental stress', *The Plant Journal*, 48(4), pp. 535–547. Available at: https://doi.org/10.1111/j.1365-313X.2006.02889.x.

Niu, M. *et al.* (2024) 'The MIR6445- *NAC029* module regulates drought tolerance by regulating the expression of glutathione S-transferase U23 and reactive oxygen species scavenging in *Populus*', *New Phytologist*, 242(5), pp. 2043–2058. Available at: https://doi.org/10.1111/nph.19703.

Ohme-Takagi, M. and Shinshi, H. (1995) 'Ethylene-Inducible DNA Binding Proteins That Interact with an Ethylene-Responsive Element', *The Plant Cell*, 7, pp. 173–182. Available at: https://doi.org/10.1105/tpc.7.2.173.

Pérez-Alonso, M.-M. *et al.* (2021) 'Jasmonic Acid-Dependent MYC Transcription Factors Bind to a Tandem G-Box Motif in the YUCCA8 and YUCCA9 Promoters to Regulate Biotic Stress Responses', *International Journal of Molecular Sciences*, 22(18), p. 9768. Available at: https://doi.org/10.3390/ijms22189768.

Pitzschke, A. *et al.* (2009) 'A Major Role of the MEKK1–MKK1/2–MPK4 Pathway in ROS Signalling', *Molecular Plant*, 2(1), pp. 120–137. Available at: https://doi.org/10.1093/mp/ssn079.

Porta, H. *et al.* (1999) 'Analysis of Lipoxygenase mRNA Accumulation in the Common Bean (Phaseolus vulgaris L.) during Development and under Stress Conditions', *Plant and Cell Physiology*, 40(8), pp. 850–858. Available at: https://doi.org/10.1093/oxfordjournals.pcp.a029614.

Qin, H. *et al.* (2023) 'Abscisic acid promotes auxin biosynthesis to inhibit primary root elongation in rice', *Plant Physiology*, 191(3), pp. 1953–1967. Available at: https://doi.org/10.1093/plphys/kiac586.

Raju, S. (2008) 'Comparative study on the induction of defense related enzymes in two different cultivars of chickpea (Cicer arietinum L) genotypes by salicylic acid, spermine and Fusarium oxysporum f. sp. ciceri', *Australian Journal of Crop Science*, 2(3), pp. 121–140.

Rasheed, S. *et al.* (2016) 'Transcriptomic Analysis of Soil-Grown Arabidopsis thaliana Roots and Shoots in Response to a Drought Stress', *Frontiers in Plant Science*, 7. Available at: https://doi.org/10.3389/fpls.2016.00180.

Rasheed, S. *et al.* (2018) 'The modulation of acetic acid pathway genes in Arabidopsis improves survival under drought stress', *Scientific Reports*, 8(1), p. 7831. Available at: https://doi.org/10.1038/s41598-018-26103-2.

Rizhsky, L. *et al.* (2004) 'When Defense Pathways Collide. The Response of Arabidopsis to a Combination of Drought and Heat Stress', *Plant Physiology*, 134(4), pp. 1683–1696. Available at: https://doi.org/10.1104/pp.103.033431.

Saeng-ngam, S. *et al.* (2012) 'The role of the OsCam1-1 salt stress sensor in ABA accumulation and salt tolerance in rice', *Journal of Plant Biology*, 55(3), pp. 198–208. Available at: https://doi.org/10.1007/s12374-011-0154-8.

Sáez-Vásquez, J., Gallois, P. and Delseny, M. (2000) 'Accumulation and nuclear targeting of BnC24, a Brassica napus ribosomal protein corresponding to a mRNA accumulating in response to cold treatment', *Plant Science*, 156(1), pp. 35–46. Available at: https://doi.org/10.1016/S0168-9452(00)00229-6.

Safavi-Rizi, V. *et al.* (2024) 'Reciprocal modulation of responses to nitrate starvation and hypoxia in roots and leaves of *Arabidopsis thaliana*', *Plant Signaling & Behavior*, 19(1), p. 2300228. Available at: https://doi.org/10.1080/15592324.2023.2300228.

Saito, S. *et al.* (2004) 'Arabidopsis *CYP707A* s Encode (+)-Abscisic Acid 8′-Hydroxylase, a Key Enzyme in the Oxidative Catabolism of Abscisic Acid', *Plant Physiology*, 134(4), pp. 1439–1449. Available at: https://doi.org/10.1104/pp.103.037614.

Sakuraba, Y. *et al.* (2015) 'The Arabidopsis Transcription Factor NAC016 Promotes Drought Stress Responses by Repressing *AREB1* Transcription through a Trifurcate Feed-Forward Regulatory Loop Involving NAP', *The Plant Cell*, 27(6), pp. 1771–1787. Available at: https://doi.org/10.1105/tpc.15.00222.

Santaniello, A. *et al.* (2014) 'A reassessment of the role of sucrose synthase in the hypoxic sucrose-ethanol transition in A rabidopsis', *Plant, Cell & Environment*, 37(10), pp. 2294–2302. Available at: https://doi.org/10.1111/pce.12363.

Schippers, J.H.M. *et al.* (2024) 'ERFVII -controlled hypoxia responses are in part facilitated by MEDIATOR SUBUNIT 25 in *Arabidopsis thaliana*', *The Plant Journal*, 120(2), pp. 748–768. Available at: https://doi.org/10.1111/tpj.17018.

Seo, P.J. *et al.* (2011) 'An Arabidopsis senescence-associated protein SAG29 regulates cell viability under high salinity', *Planta*, 233(1), pp. 189–200. Available at: https://doi.org/10.1007/s00425-010-1293-8.

Seok, H.-Y. *et al.* (2017) 'Arabidopsis AtNAP functions as a negative regulator via repression of AREB1 in salt stress response', *Planta*, 245(2), pp. 329–341. Available at: https://doi.org/10.1007/s00425-016-2609-0.

Seong, E.S. *et al.* (2007) 'Induction of enhanced tolerance to cold stress and disease by overexpression of the pepper *CaPIF1* gene in tomato', *Physiologia Plantarum*, 129(3), pp. 555–566. Available at: https://doi.org/10.1111/j.1399-3054.2006.00839.x.

Sofo, A. *et al.* (2004) 'Lipoxygenase activity and proline accumulation in leaves and roots of olive trees in response to drought stress', *Physiologia Plantarum*, 121(1), pp. 58–65. Available at: https://doi.org/10.1111/j.0031-9317.2004.00294.x.

Son, G.H. *et al.* (2012) 'Ethylene-Responsive Element-Binding Factor 5, ERF5, Is Involved in Chitin-Induced Innate Immunity Response', *Molecular Plant-Microbe Interactions®*, 25(1), pp. 48–60. Available at: https://doi.org/10.1094/MPMI-06-11-0165.

Song, J. *et al.* (2014) 'The rice nuclear gene WLP1 encoding a chloroplast ribosome L13 protein is needed for chloroplast development in rice grown under low temperature conditions', *Plant Molecular Biology*, 84(3), pp. 301–314. Available at: https://doi.org/10.1007/s11103-013-0134-0.

Srivastava, A.K. *et al.* (2009) 'Thiourea modulates the expression and activity profile of mtATPase under salinity stress in seeds of Brassica juncea', *Annals of Botany*, 103(3), pp. 403–410. Available at: https://doi.org/10.1093/aob/mcn229.

Stief, A. *et al.* (2014) '*Arabidopsis miR156* Regulates Tolerance to Recurring Environmental Stress through *SPL* Transcription Factors', *The Plant Cell*, 26(4), pp. 1792–1807. Available at: https://doi.org/10.1105/tpc.114.123851.

Sun, C.-H., Yang, C.-Y. and Tzen, J.T.C. (2018) 'Molecular Identification and Characterization of Hydroxycinnamoyl Transferase in Tea Plants (Camellia sinensis L.)', *International Journal of Molecular Sciences*, 19(12), p. 3938. Available at: https://doi.org/10.3390/ijms19123938.

Survila, M. *et al.* (2016) 'Peroxidase-Generated Apoplastic ROS Impair Cuticle Integrity and Contribute to DAMP-Elicited Defenses', *Frontiers in Plant Science*, 7. Available at: https://doi.org/10.3389/fpls.2016.01945.

Suzuki, N. *et al.* (2005) 'Enhanced Tolerance to Environmental Stress in Transgenic Plants Expressing the Transcriptional Coactivator Multiprotein Bridging Factor 1c', *Plant Physiology*, 139(3), pp. 1313–1322. Available at: https://doi.org/10.1104/pp.105.070110.

Szechyńska-Hebda, M. *et al.* (2016) 'PAD4, LSD1 and EDS1 regulate drought tolerance, plant biomass production, and cell wall properties', *Plant Cell Reports*, 35(3), pp. 527–539. Available at: https://doi.org/10.1007/s00299-015-1901-y.

Tahir, M.S. (2022) 'HD2A and HD2C co-regulate drought stress response by modulating stomatal closure and root growth in Arabidopsis', *Frontiers in Plant Science*, 13, 1062722. Available at: https://doi.org/10.3389/fpls.2022.1062722

Tang, H. *et al.* (2021) 'WRKY33 interacts with WRKY12 protein to up-regulate *RAP2 . 2* during submergence induced hypoxia response in *Arabidopsis thaliana*', *New Phytologist*, 229(1), pp. 106–125. Available at: https://doi.org/10.1111/nph.17020.

Thipyapong, P. *et al.* (2004) 'Suppression of polyphenol oxidases increases stress tolerance in tomato', *Plant Science*, 167(4), pp. 693–703. Available at: https://doi.org/10.1016/j.plantsci.2004.04.008.

To, T.K. *et al.* (2011) 'Arabidopsis HDA6 is required for freezing tolerance', *Biochemical and Biophysical Research Communications*, 406(3), pp. 414–419. Available at: https://doi.org/10.1016/j.bbrc.2011.02.058.

Tran, L.-S.P. *et al.* (2004) ‘Isolation and Functional Analysis of Arabidopsis Stress-Inducible NAC Transcription Factors That Bind to a Drought-Responsive *cis* -Element in the *early responsive to dehydration stress 1* Promoter[W]’, *The Plant Cell*, 16(9), pp. 2481–2498. Available at: https://doi.org/10.1105/tpc.104.022699.

Wang, C.-S., Hsu, S.-W. and Hsu, Y.-F. (2013) ‘New Insights into Desiccation-Associated Gene Regulation by Lilium longiflorum ASR during Pollen Maturation and in Transgenic Arabidopsis’, in *International Review of Cell and Molecular Biology*. Elsevier, pp. 37–94. Available at: https://doi.org/10.1016/B978-0-12-407704-1.00002-6.

Wang, T. *et al.* (2015) ‘Salt-Related MYB1 Coordinates Abscisic Acid Biosynthesis and Signaling during Salt Stress in Arabidopsis’, *Plant Physiology*, 169(2), pp. 1027–1041. Available at: https://doi.org/10.1104/pp.15.00962.

Wang, Y. *et al.* (2019) ‘iTRAQ-Based Quantitative Analysis of Responsive Proteins Under PEG-Induced Drought Stress in Wheat Leaves’, *International Journal of Molecular Sciences*, 20(11), p. 2621. Available at: https://doi.org/10.3390/ijms20112621.

Wang, Y. *et al.* (2023) ‘Genome-Wide Analysis of the Rad21/REC8 Gene Family in Cotton (Gossypium spp.)’, *Genes*, 14(5), p. 993. Available at: https://doi.org/10.3390/genes14050993.

Weng, M. *et al.* (2014) ‘Histone chaperone ASF1 is involved in gene transcription activation in response to heat stress in *Arabidopsis thaliana*’, *Plant, Cell & Environment*, 37(9), pp. 2128–2138. Available at: https://doi.org/10.1111/pce.12299.

Wituszyńska, W. *et al.* (2013) ‘LESION SIMULATING DISEASE1, ENHANCED DISEASE SUSCEPTIBILITY1, and PHYTOALEXIN DEFICIENT4 Conditionally Regulate Cellular Signaling Homeostasis, Photosynthesis, Water Use Efficiency, and Seed Yield in Arabidopsis’, *Plant Physiology*, 161(4), pp. 1795–1805. Available at: https://doi.org/10.1104/pp.112.208116.

Xu, J. *et al.* (2008) ‘Activation of MAPK Kinase 9 Induces Ethylene and Camalexin Biosynthesis and Enhances Sensitivity to Salt Stress in Arabidopsis’, *Journal of Biological Chemistry*, 283(40), pp. 26996–27006. Available at: https://doi.org/10.1074/jbc.M801392200.

Xu, R. *et al.* (2016) ‘Global identification and expression analysis of stress-responsive genes of the Argonaute family in apple’, *Molecular Genetics and Genomics*, 291(6), pp. 2015–2030. Available at: https://doi.org/10.1007/s00438-016-1236-6.

Yamaguchi, N. *et al.* (2021) ‘H3K27me3 demethylases alter HSP22 and HSP17.6C expression in response to recurring heat in Arabidopsis’, *Nature Communications*, 12(1), p. 3480. Available at: https://doi.org/10.1038/s41467-021-23766-w.

Yang, C. *et al.* (2023) ‘PIF7-mediated epigenetic reprogramming promotes the transcriptional response to shade in *Arabidopsis*’, *The EMBO Journal*, 42(8), p. e111472. Available at: https://doi.org/10.15252/embj.2022111472.

Yang, J. *et al.* (2020) ‘Expansin gene TaEXPA2 positively regulates drought tolerance in transgenic wheat (Triticum aestivum L.)’, *Plant Science*, 298, p. 110596. Available at: https://doi.org/10.1016/j.plantsci.2020.110596.

Yang, L. *et al.* (2013) ‘Expression of a wild eggplant ribosomal protein L13a in potato enhances resistance to Verticillium dahliae’, *Plant Cell, Tissue and Organ Culture (PCTOC)*, 115(3), pp. 329–340. Available at: https://doi.org/10.1007/s11240-013-0365-4.

Yang, W.-J. *et al.* (2019) ‘Overexpression of TaCOMT Improves Melatonin Production and Enhances Drought Tolerance in Transgenic Arabidopsis’, *International Journal of Molecular Sciences*, 20(3), p. 652. Available at: https://doi.org/10.3390/ijms20030652.

Yang, Y. *et al.* (2018) ‘RNA interference of NtNCED3 reduces drought tolerance and impairs plant growth through feedback regulation of isoprenoids in Nicotiana tabacum’, *Environmental and*

Experimental Botany, 155, pp. 332–344. Available at: https://doi.org/10.1016/j.envexpbot.2018.07.016.

Ye, H. *et al.* (2009) ‘Identification and expression profiling analysis of TIFY family genes involved in stress and phytohormone responses in rice’, *Plant Molecular Biology*, 71(3), pp. 291–305. Available at: https://doi.org/10.1007/s11103-009-9524-8.

Yeh, S.-H. *et al.* (2011) ‘Analysis of the expression of BohLOL1, which encodes an LSD1-like zinc finger protein in Bambusa oldhamii’, *Planta*, 234(6), pp. 1179–1189. Available at: https://doi.org/10.1007/s00425-011-1467-z.

Yu, X. *et al.* (2023) ‘FLS2-RBOHD module regulates changes in the metabolome of *Arabidopsis* in response to abiotic stress’, *Plant-Environment Interactions*, 4(1), pp. 36–54. Available at: https://doi.org/10.1002/pei3.10101.

Yuenyong, W. *et al.* (2019) ‘Isocitrate lyase plays important roles in plant salt tolerance’, *BMC Plant Biology*, 19(1), p. 472. Available at: https://doi.org/10.1186/s12870-019-2086-2.

Zamora-Briseño, J.A. and De Jiménez, E.S. (2016) ‘A LEA 4 protein up-regulated by ABA is involved in drought response in maize roots’, *Molecular Biology Reports*, 43(4), pp. 221–228. Available at: https://doi.org/10.1007/s11033-016-3963-5.

Zhang, J.-Y. *et al.* (2015) ‘De novo transcriptome sequencing and comparative analysis of differentially expressed genes in kiwifruit under waterlogging stress’, *Molecular Breeding*, 35(11), p. 208. Available at: https://doi.org/10.1007/s11032-015-0408-0.

Zhang, K. *et al.* (2019) ‘Overexpression of CrCOMT from Carex rigescens increases salt stress and modulates melatonin synthesis in Arabidopsis thaliana’, *Plant Cell Reports*, 38(12), pp. 1501–1514. Available at: https://doi.org/10.1007/s00299-019-02461-7.

Zhang, Q. *et al.* (2021) ‘The Responses of the Lipoxygenase Gene Family to Salt and Drought Stress in Foxtail Millet (Setaria italica)’, *Life*, 11(11), p. 1169. Available at: https://doi.org/10.3390/life11111169.

Zhao, C. *et al.* (2007) ‘Constitutive expression of an endoplasmic reticulum small heat shock protein alleviates endoplasmic reticulum stress in transgenic tomato’, *Journal of Plant Physiology*, 164(7), pp. 835–841. Available at: https://doi.org/10.1016/j.jplph.2006.06.004.

Zhao, M. *et al.* (2012) ‘Expansins are involved in cell growth mediated by abscisic acid and indole-3-acetic acid under drought stress in wheat’, *Plant Cell Reports*, 31(4), pp. 671–685. Available at: https://doi.org/10.1007/s00299-011-1185-9.

Zhao, W. *et al.* (2023) ‘Jasmonic acid enhances osmotic stress responses by MYC2 -mediated inhibition of *protein phosphatase 2C1* and *response regulators 26* transcription factor in tomato’, *The Plant Journal*, 113(3), pp. 546–561. Available at: https://doi.org/10.1111/tpj.16067.

Zheng, X. *et al.* (2019) ‘Antiviral ARGONAUTEs Against *Turnip Crinkle Virus* Revealed by Image-Based Trait Analysis’, *Plant Physiology*, 180(3), pp. 1418–1435. Available at: https://doi.org/10.1104/pp.19.00121.

Zheng, Z. *et al.* (2006) ‘Arabidopsis WRKY33 transcription factor is required for resistance to necrotrophic fungal pathogens’, *The Plant Journal*, 48(4), pp. 592–605. Available at: https://doi.org/10.1111/j.1365-313X.2006.02901.x.

Zhu, J. *et al.* (2008) ‘Involvement of *Arabidopsis* HOS15 in histone deacetylation and cold tolerance’, *Proceedings of the National Academy of Sciences*, 105(12), pp. 4945–4950. Available at: https://doi.org/10.1073/pnas.0801029105.

Zhu, Y., Dong, A. and Shen, W.-H. (2012) ‘Histone variants and chromatin assembly in plant abiotic stress responses’, *Biochimica et Biophysica Acta (BBA) - Gene Regulatory Mechanisms*, 1819(3–4),

pp. 343–348. Available at: https://doi.org/10.1016/j.bbagrm.2011.07.012.
